# Passivation‐Induced Species Dynamics and Microstructural Evolution in Solid‐State Lithium–Sulfur Cathodes

**DOI:** 10.1002/advs.202520537

**Published:** 2026-01-26

**Authors:** Arpan K. Sharma, Bairav S. Vishnugopi, Elif Pınar Alsaç, Matthew T. McDowell, Partha P. Mukherjee

**Affiliations:** ^1^ School of Mechanical Engineering Purdue University West Lafayette Indiana USA; ^2^ George W. Woodruff School of Mechanical Engineering Georgia Institute of Technology Atlanta Georgia USA; ^3^ School of Materials Science and Engineering Georgia Institute of Technology Atlanta Georgia USA

**Keywords:** electrode microstructure, lithium–sulfur cathode, reaction kinetics, solid‐state batteries, transport limitations

## Abstract

Solid‐state lithium–sulfur (SSLS) batteries offer high theoretical energy density, yet their practical viability is hindered by poor sulfur utilization and limited rechargeability. At the core of this challenge lies the passivating nature of Li_2_S, which restricts ionic and electronic transport, suppresses interfacial activity, and severely impedes the reversibility of electrochemical reactions. In this study, we elucidate the mechanistic origins of these limitations by resolving how charge and discharge species form, grow, and spatially evolve within the cathode microstructure under varied current densities and electrode compositions. By resolving the species distribution at the particle scale and coupling it with Raman spectroscopy and X‐ray diffraction, we demonstrate how Li_2_S formation induces localized surface passivation that progressively limits electrochemical accessibility within the cathode microstructure. Sulfur utilization is found to be strongly governed by the interplay between sulfur loading, residual porosity, and interfacial architecture. High sulfur contents result in buried, electrochemically isolated domains due to poor solid electrolyte (SE) percolation, while low sulfur contents trigger SE degradation via parasitic reactions. The resulting sulfur‐porosity maps delineate the mechanistic boundaries between reversible and transport‐limited regimes, offering actionable design guidance for SSLS cathodes with enhanced sulfur utilization.

## Graphical abstract

The formation of insulating Li_2_S during discharge in solid‐state lithium–sulfur batteries passivate reaction sites and limits sulfur utilization. In this work, a microstructure‐resolved modeling framework coupling transport and reaction kinetics is developed to predict charge–discharge behavior and reveal particle‐scale species evolution and incomplete sulfur reduction and oxidation driven by Li_2_S surface passivation.

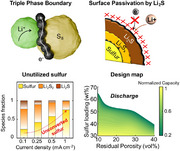

## 1 Introduction

Lithium–sulfur (Li–S) batteries are widely recognized as one of the most promising candidates among “beyond lithium‐ion” energy storage chemistries. With a high theoretical specific energy density of 2600 Wh kg^−1^, substantially surpassing the 350–400 Wh kg^−1^ range of conventional lithium‐ion systems, Li–S cells have garnered significant attention since their original patent in 1962 [1, 2, 3, 4, 5, 6]. However, their practical implementation has remained elusive, largely due to polysulfide dissolution and the resulting shuttle effect, which leads to parasitic deposition at the anode and irreversible capacity loss [7, 8, 9, 10, 11, 12, 13, 14]. To overcome these challenges, solid‐state Li–S (SSLS) batteries have emerged as a viable alternative. By replacing the liquid electrolyte with a solid electrolyte (SE), SSLS batteries effectively suppress the polysulfide shuttle phenomenon [15, 16, 17, 18, 19, 20, 21], may help impede lithium dendrite growth via the mechanical rigidity of SEs [22, 23, 24], and improve safety by eliminating flammable liquid components [25, 26, 27, 28, 29, 30, 31].

**FIGURE 1:**
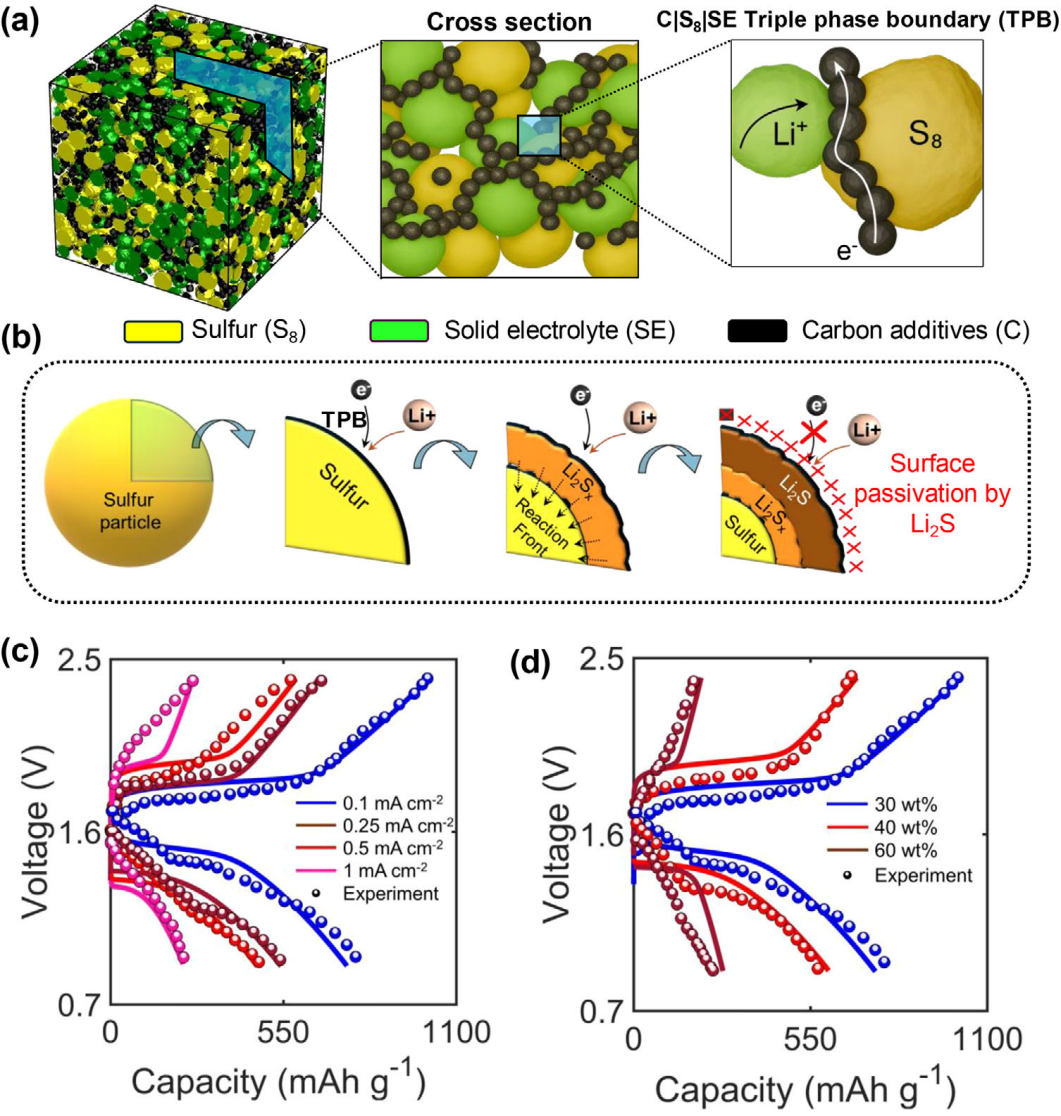
(a) Schematic of the multiphase cathode architecture comprising sulfur, SE, and carbon additives, with electrochemical reactions occurring at the triple‐phase boundaries. (b) Stepwise discharge pathway illustrating the reduction of sulfur to Li_2_S_x_ intermediates and final formation of insulating Li_2_S. (c) Comparison of the electrochemical response of SSLS cells predicted by the mathematical model against the experimental values at (c) 30 wt.% sulfur loading with four different current densities of 0.1, 0.25, 0.5 and 1 mA cm^−2^, and sulfur loadings of (a) 30 wt.%, (b) 40 wt.%, and (c) 60 wt.% at a current density of 0.1 mA cm^−2^.

Figure 1a illustrates a schematic of a typical composite cathode in a SSLS cell, comprising elemental sulfur (S_8_), SE, and carbon additives such as acetylene black (AB), which compensate for the electronically insulating nature of sulfur and its discharge products. Unlike in liquid electrolyte‐based Li–S systems, where the electrolyte percolates through the porous electrode and uniformly wets sulfur surfaces, SEs establish point‐to‐point contacts between SE‐SE and SE‐sulfur particles [32]. These localized interfaces support Li^+^ transport and define the reactive C|S_8_|SE interface essential for electrochemical processes (Figure 1a). The effectiveness of such transport and interfacial reactions is highly sensitive to the cathode microstructure. Voids formed during fabrication can interrupt these percolation pathways, limiting both ionic mobility and reaction kinetics [33, 34]. These structural constraints not only hinder species transport but also critically influence the progression, localization, and reversibility of electrochemical transformations within the cathode.

In a liquid electrolyte‐based Li–S cell, sulfur dissolves during discharge to form polysulfides of varying chain lengths, which subsequently precipitate as Li_2_S [9, 10, 35, 36, 37, 38, 39, 40, 41]. In contrast, SSLS cells exhibit a direct solid‐solid conversion of sulfur with Li^+^, forming Li_2_S at the cathode without any dissolution or precipitation. During charging, the reverse reaction proceeds with Li_2_S converting back to sulfur via the formation of intermediate polysulfides. However, the detailed reaction mechanism in SSLS cathodes remains under active investigation.

While early studies suggested a direct sulfur‐to‐Li_2_S conversion [42], recent works have revealed a more complex pathway involving metastable intermediates such as Li_2_S_2_, Li_2_S_4_, and Li_2_S_8_ [43, 44]. Operando analyses and theoretical studies have confirmed that the discharge product is not exclusively Li_2_S but a mixture of Li_2_S and Li_2_S_2_ [43, 44, 45, 46, 47, 48]. Kim et al. [45] employed synchrotron X‐ray absorption spectroscopy (XAS) and X‐ray photoelectron spectroscopy (XPS) to confirm the presence of mixed Li_2_S_2_/Li_2_S discharge products. Similar spectral features were reported by Cao et al. [44], indicating Li_2_S_2_ accumulation due to sluggish reaction kinetics in SSLS cathodes. The metastable nature of Li_2_S_2_, coupled with its structural resemblance to Li_2_S and overlapping spectral features, makes its detection particularly challenging [45]. As the discharge progresses, Li_2_S emerges as the dominant product; however, its poor ionic and electronic conductivity results in the formation of a passivating layer around sulfur particles, effectively stalling further redox progression and limiting active material utilization (Figure 1b).

Although the discharge process proceeds via successive reduction, the reversibility of this pathway during charging remains less certain. Gu et al. [49] reported a one‐step Li_2_S‐to‐sulfur conversion during charging at elevated temperature, suggesting a kinetically driven transformation. In contrast, Cao et al. [44] detected Li_2_S_2_ even during charging using operando Raman spectroscopy and ex situ XAS. Furthermore, the reconversion of Li_2_S to sulfur is severely hindered by its electrochemical inactivity, resulting in substantial activation overpotentials that inhibit full oxidation. This kinetic bottleneck is a key limitation to the practical reversibility of SSLS cells [45, 50, 51, 52, 53]. This underscores the critical need to elucidate the passivation effects of Li_2_S and their direct impact on cell‐level performance limitations.

To gain mechanistic insight into these dynamics, modeling frameworks, when coupled with experimental studies, offer a powerful tool to probe species evolution and microstructural changes during cycling. Yin and Franco [54] pioneered a kinetic model to describe discharge in quasi‐solid‐state Li–S batteries via a two‐step pathway involving a Li_2_S_2_ intermediate. However, their approach remains constrained by the presence of a liquid phase, limiting its relevance to fully solid‐state systems. This gap underscores the need for advanced physics‐based models that incorporate the distinctive physicochemical features of SSLS batteries to enable more accurate performance predictions across diverse operating regimes.

In this work, we develop a comprehensive microstructure‐resolved modeling framework for SSLS batteries that couples transport phenomena and reaction kinetics to predict charge–discharge behavior. The model uncovers key mechanistic features of species evolution at the particle scale and elucidates the incomplete reduction and oxidation of sulfur due to Li_2_S passivation. We track the dynamic coexistence of different species under varying conditions and relate these pathways to cell‐level electrochemical performance. Through parametric analysis of sulfur loading and residual porosity, we identify how competing kinetic and transport limitations manifest across different cathode architectures, yielding design principles for optimized microstructures. To complement the modeling, we employ Raman spectroscopy and X‐ray diffraction (XRD) on cathodes composed of sulfur, Li_6_PS_5_Cl (LPSCl) as the SE, and AB to investigate intermediate species formation and structural evolution during cell operation. Overall, this integrated modeling‐experimental framework provides a mechanistic bridge between species‐scale electrochemistry and macroscale performance in SSLS cells.

## 2 Results and Discussion

We investigate the electrochemical behavior of a representative SSLS cell with a composite cathode consisting of 30 wt.% sulfur (5 µm), 50 wt.% LPSCl (1 µm) as SE, and 20 wt.% AB as the conductive additive, coupled with a Li–In alloy counter electrode. The solid‐solid conversion reaction is modeled through a physics‐based mesoscale framework that explicitly resolves both phase transition kinetics and microstructural disparities. In line with literature evidence [43, 44, 45, 46, 47], the reaction proceeds via a two‐step pathway: sulfur first reduces to Li_2_S_2_ and subsequently to Li_2_S, with distinct Butler–Volmer kinetics assigned to each step to capture the faster conversion to Li_2_S_2_ and the slower, rate‐limiting transformation to Li_2_S (see Section S1). The reaction currents are defined as:

(1) $$\begin{array}{c} i_{1}=n_{1}Fk_{1}\left\{\;\varepsilon_{S_{8}}^{*}\exp\left(\beta_{1}\frac{F}{RT}\eta_{1}\right)-\varepsilon_{Li_{2}S_{2}}^{*}\exp\left(-\left(1-\beta_{1}\right)\frac{F}{RT}\eta_{1}\right)\;\right\}\;\;\;\;\;\; \end{array}$$

(2) $$\eta_{1}=\phi_{S}-\phi_{E}-E_{1}^{0}$$

(3) $$\begin{array}{c} i_{2}=n_{2}Fk_{2}\left\{\;\varepsilon_{Li_{2}S_{2}}^{*}\exp\left(\beta_{2}\frac{F}{RT}\eta_{2}\;\right)-\varepsilon_{Li_{2}S}^{*}\exp\left(\;-\left(1-\beta_{2}\right)\frac{F}{RT}\eta_{2}\;\right)\;\;\right\}\;\;\;\;\; \end{array}$$

(4) $$\eta_{2}=\phi_{S}-\phi_{E}-E_{2}^{0}$$

Here, η_1_ and η_2_ are the overpotentials that arise from the difference between the local solid‐phase potential ϕ_*S*_, the electrolyte potential ϕ_*e*_, and the respective equilibrium potentials E^0^. This formulation ensures that voltage polarization, arising from the coupled ionic and electronic transport in the composite cathode, governs the reaction kinetics and phase transformations. To represent interfacial kinetic limitations, the model further incorporates a surface passivation penalty that links the active interfacial area to Li_2_S accumulation, thereby reproducing the insulating effect of Li_2_S and the associated rise in overpotentials during cycling. In parallel, microstructural heterogeneity is addressed through stochastically generated cathode architectures, from which tortuosity, electronic conductivity, and active interfacial area are quantified using direct numerical simulations. These descriptors are directly integrated into the electrochemical equations, ensuring that the influence of microstructure on transport and reaction kinetics is accurately represented. Additional details of the reaction pathway, governing equations, and property calculations are described in Section S1. To isolate the effect of surface passivation on electrochemical performance, the present framework assumes a fixed cathode geometry and does not account for cathode volume expansion or chemo‐mechanical deformation arising from sulfur conversion.

The predictive capability of this framework is demonstrated through extensive validation against experimental data across multiple sulfur loadings and current densities (Figure 1c,d). The strong agreement between simulated and measured charge‐discharge profiles confirms that the model captures the highly nonlinear electrochemical response of SSLS cells, including the interplay of transport limitations, interfacial kinetics and surface passivation.

**FIGURE 2:**
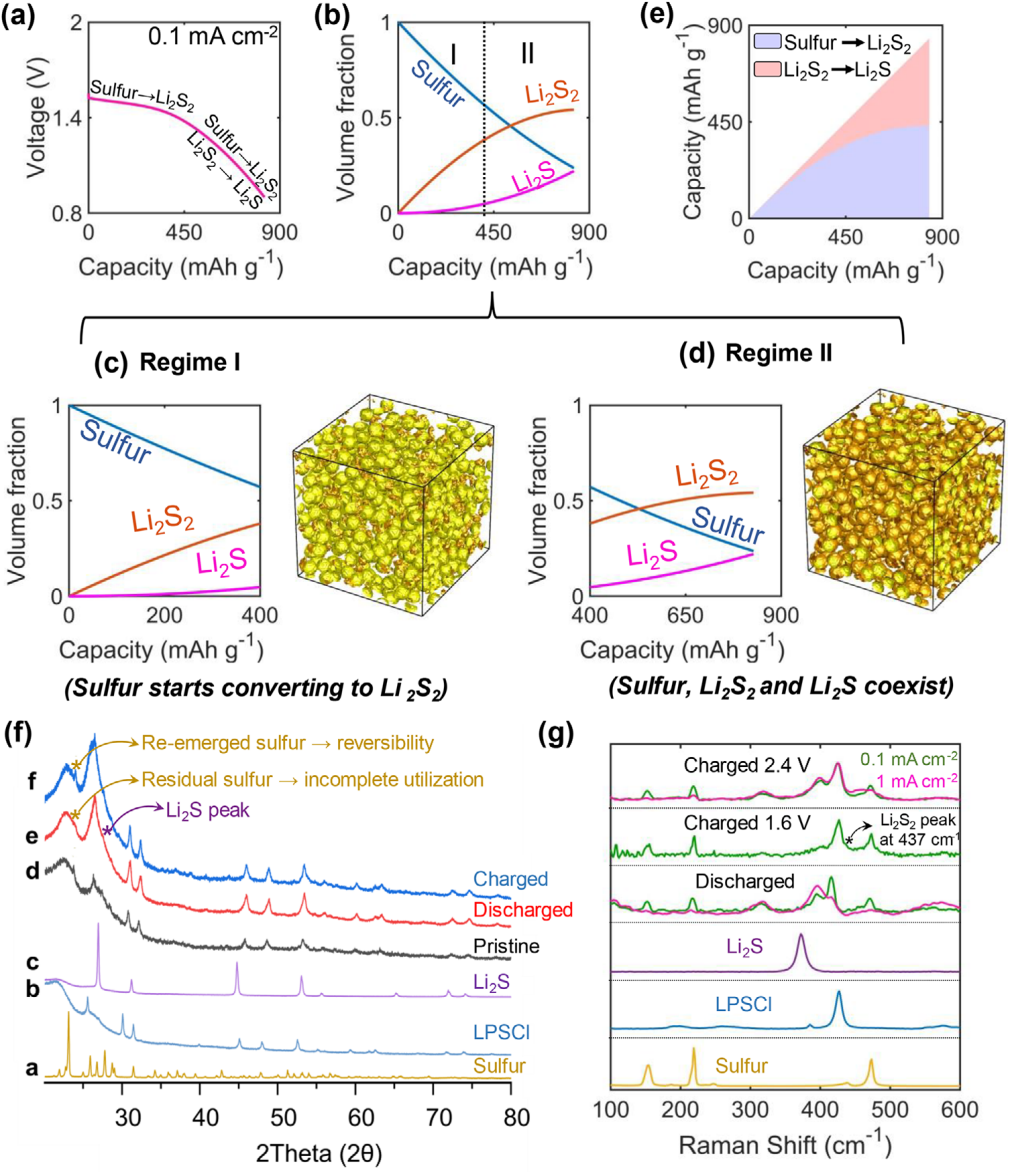
Simulation results for the SSLS cell with a Li–In counter electrode and 30 wt.% sulfur loading: (a) discharge voltage profile; (b) species evolution during discharge, revealing two distinct regimes; (c) Regime I: initial conversion of sulfur to Li_2_S_2_; (d) Regime II: coexistence of sulfur, Li_2_S_2_, and Li_2_S; (e) capacity contributions from the sequential steps: sulfur → Li_2_S_2_ and Li_2_S_2_ → Li_2_S. (f) XRD patterns of (a) sulfur, (b) LPSCl, (c) Li_2_S precursors and 30% sulfur composite cathodes at (d) pristine, (e) discharge and (f) charged states. (g) Raman spectra at different states of charge and tested at different current densities, highlighting the emergence of the Li_2_S_2_ peak at 437 cm^−1^ for different control samples.

Figure 2a shows the simulated discharge behavior, with the cell delivering a specific capacity of 839 mAh g^−1^ at a current density of 0.1 mA cm^−2^. The discharge proceeds via sequential sulfur reduction: first to Li_2_S_2_, followed by conversion to Li_2_S. Owing to its comparatively higher ionic and electronic conductivity, Li_2_S_2_ undergoes faster redox kinetics than Li_2_S [43, 45, 54]. In contrast, Li_2_S is highly insulating, and its accumulation on the particle surfaces impedes both electron and Li^+^ transport, making the Li_2_S_2_ → Li_2_S conversion the rate‐limiting step. The discharge curve features an initial plateau around 1.50 V, which corresponds to the equilibrium potential of sulfur reduction to Li_2_S_2_ and marks the onset of this transformation. The species evolution (Figure 2b) reveals two distinct regimes: In Regime I, sulfur is predominantly converted to Li_2_S_2_, which forms as a kinetically stabilized intermediate with negligible Li_2_S production (Figure 2c); In Regime II, Li_2_S_2_ begins converting to Li_2_S while unreacted sulfur continues reducing to Li_2_S_2_ (Figure 2d).

As Li_2_S accumulates, it passivates active cathode surfaces, forming a shell around sulfur particles that impedes further reaction (see Surface passivation, Section S1.3). This progressive passivation manifests as an increased kinetic overpotential and a sharper voltage drop in the later stages of discharge (Figure 2a). As a consequence of this passivation, the conversion reactions remain incomplete with approximately 23% of sulfur and 54% of Li_2_S_2_ remain unreacted at the end of discharge (Figure 2b,d). It is important to note that species concentrations are expressed in terms of sulfur content, and the combined total always sums to 100%, ensuring sulfur mass conservation (see Section S1). Of the total 839 mAh g^−1^ capacity, the contribution from the sulfur → Li_2_S_2_ reaction slightly exceeds that from Li_2_S_2_ → Li_2_S (430 vs. 409 mAh g^−1^), consistent with the incomplete conversion of Li_2_S_2_ (Figure 2e).

To confirm the presence of unconverted sulfur, we performed XRD analysis of the cathode composites. In the discharged samples, sulfur peaks are noticeably diminished, indicating substantial sulfur consumption during discharge; however, the persistence of these weakened peaks confirms that a portion of sulfur remains unutilized (Figure 2f). Additionally, weak (111) reflections corresponding to Li_2_S are observed in the discharged state, suggesting the presence of Li_2_S as part of the discharge product and indicating its poorly crystalline nature. Upon charging, the reappearance of distinct sulfur peaks confirms the reconversion of Li_2_S to sulfur, indicating the (at least partial) reversibility of the overall conversion process, which is further discussed in a later section.

Raman spectroscopy was further conducted on pristine, discharged, and charged electrode samples, as well as on the constituent materials (Figure 2g). For reference, Raman spectra of the individual constituents show distinct peaks for sulfur (158, 220, and 473 cm^−1^), LPSCl (199, 268, 430, 574, and 599 cm^−1^), and Li_2_S (374 cm^−1^). In the pristine composite electrode, only the sulfur and LPSCl peaks are present. During discharge, sulfur is expected to convert to Li_2_S; however, the Raman spectrum of the discharged sample reveals only a weak and broad shoulder at 375 cm^−1^, indicating the presence of largely amorphous Li_2_S. No clear peaks corresponding to intermediate polysulfides are detected in either the fully discharged or charged samples. This absence may be attributed to the metastable and/or transient nature of these species, which often evade experimental detection. To further examine the presence of intermediates, ex situ Raman analysis was performed on a cell held at 1.6 V during charging, revealing a distinct small peak at 437 cm^−1^ attributable to Li_2_S_2_ (Figure 2g). This observation aligns with earlier reports of Li_2_S_2_ formation during solid‐solid conversion and supports our modeling assumptions [44, 55]. Additionally, all cycled samples exhibited a peak near 390 cm^−1^, attributed to P_2_S_7_^4−^ chain formation [56, 57, 58], indicating partial degradation of LPSCl. This degradation may occur during both ball‐milling and electrochemical cycling. Together, XRD and Raman analyses reveal incomplete sulfur utilization, marked by amorphous and/or poorly crystalline Li_2_S formation and transient Li_2_S_2_ intermediates. These features indicate a redox pathway constrained by sluggish kinetics and limited transport.

**FIGURE 3:**
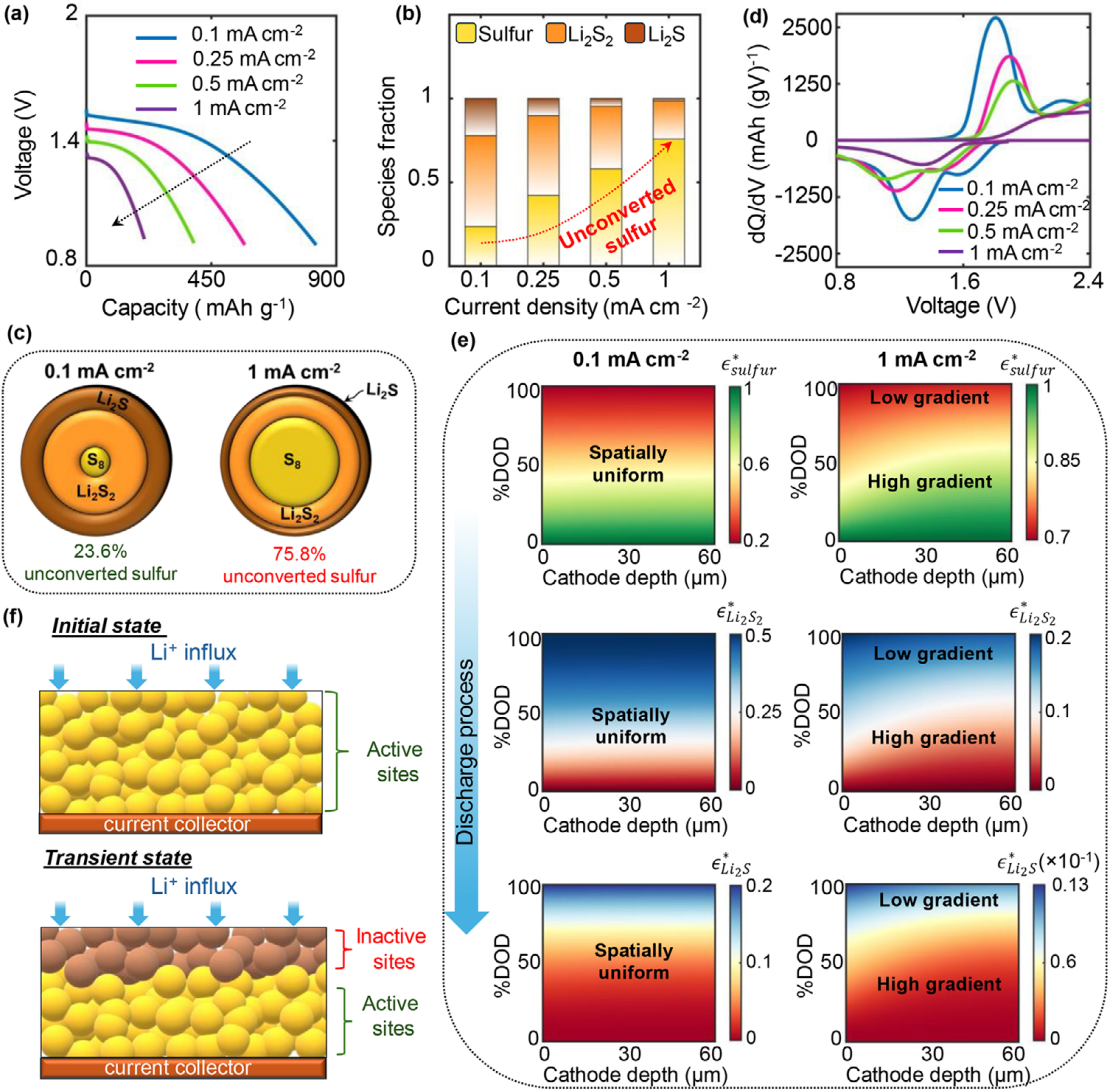
Simulation results showing (a) discharge voltage profiles at varying current densities (0.1–1 mA cm^−2^); (b) species fraction analysis showing increased unconverted sulfur at higher current densities. (c) Schematic illustrating insulating Li_2_S shell formation around sulfur particles, hindering complete conversion. (d) Experimental differential capacity (dQ/dV) curves as a function of current density illustrating current‐dependent shifts in redox features using Li–In/Li counter electrode. (e) Simulated spatio‐temporal distribution of sulfur (top), Li_2_S_2_ (middle), and Li_2_S (bottom) across the cathode depth as a function of depth of discharge (DOD), shown for two discharge rates: 0.1 mA cm^−2^ (left) and 1 mA cm^−2^ (right). The downward direction corresponds to discharge species forming during the reaction, while the horizontal direction compares the same species at the two discharge rates. High‐rate discharge leads to pronounced concentration gradients near the cathode‐separator interface, which diminishes toward the end of discharge due to reaction current redistribution. (f) Schematic depicting loss of active sites near the separator‐cathode interface due to Li_2_S accumulation, causing current redistribution deeper into the cathode.

These limitations become more pronounced at higher current densities. For example, the model predicts a 74% reduction in capacity with a ten‐fold increase in current density, from 839 mAh g^−1^ at 0.1 mA cm^−2^ to just 215 mAh g^−1^ at 1 mA cm^−2^ (Figure 3a). This capacity loss is primarily attributed to increased transport and kinetic overpotentials in the cathode, arising from sluggish Li^+^ transport and the rapid formation of an insulating Li_2_S layer over sulfur particles. At 0.1 mA cm^−2^, the fractions of unconverted sulfur and Li_2_S_2_ are 23% and 54%, respectively, with 23% conversion to Li_2_S (Figure 3b). The low current density allows gradual formation of the Li_2_S shell, facilitating continued conversion of sulfur to Li_2_S_2_ and subsequently to Li_2_S. However, once the particle surface is fully coated by Li_2_S, electron and Li^+^ access to the interior is blocked, leaving the core unreacted (Figure 3c).

As the discharge rate increases, Li_2_S forms more rapidly, accelerating surface passivation and exacerbating kinetic overpotential. This is compounded by limited Li^+^ transport within the cathode. At a current density of 1 mA cm^−2^, sulfur utilization is drastically reduced, with 88% of sulfur remaining unconverted and only 1.3% forming Li_2_S, resulting in the steep capacity drop to 215 mAh g^−1^. The rapid formation of the insulating Li_2_S film effectively blocks active sites and imposes severe kinetic limitations, reflected in the sharp voltage drop (Figure 3a).

These trends are further supported by current‐dependent experimental dQ/dV curves (Figure 3d). At lower current densities, the curves display a distinct peak (at 1.3 V) and shoulder (at 1.6 V), features commonly associated with multi‐step sulfur reduction processes. In contrast, higher current densities lead to diminished and merged features, shifted toward lower potentials during discharge and higher potentials during charge. This shift reflects increased kinetic limitations and polarization, primarily arising from sluggish Li^+^ transport and the rapid formation of electrochemically inactive Li_2_S. The voltage behavior during charging is discussed in detail in a later section. These electrochemical signatures not only reflect reaction kinetics but also hint at spatial heterogeneities in species evolution within the cathode, especially under rate‐limited conditions.

While the Raman spectroscopy and XRD results confirm the presence of sulfur, Li_2_S_2_ and Li_2_S and validate the qualitative trends in species evolution, the absolute quantification of phase fractions in dense solid‐state composites experimentally remains a significant challenge. The intrinsic heterogeneity of SSLSs electrodes, coupled with overlapping spectral signatures and the amorphous nature of discharge products, limits the ability of standard laboratory techniques to reliably yield molar phase fractions. Even advanced spectroscopic approaches, such as synchrotron‐based X‐ray absorption spectroscopy and ToF‐SIMS, are primarily utilized to infer relative phase compositions rather than absolute sulfur utilization [45]. Consequently, the physics‐based mesoscale framework developed in this work serves as a necessary tool to provide the quantitative spatial and temporal distributions of species that are not directly accessible through conventional experiments. The development of new operando methodologies for absolute molar quantification remains essential for the field.

The evolution of species distribution across the cathode is strongly influenced by the interplay between local reaction kinetics and ionic transport limitations. At a low current density of 0.1 mA cm^−2^, Li^+^ transport is sufficiently fast to match the reaction kinetics. As a result, sulfur reduction proceeds relatively uniformly throughout the electrode thickness, yielding spatially homogeneous evolution of sulfur, Li_2_S_2_, and Li_2_S, with minimal concentration gradients (Figure 3e). In contrast, at a higher discharge rate of 1 mA cm^−2^, Li^+^ transport becomes insufficient to sustain the increased electrochemical demand, particularly in deeper regions of the electrode farther from the separator. Consequently, sulfur reduction is concentrated near the cathode‐separator interface, where Li^+^ availability is highest, resulting in sharp concentration gradients of species along the electrode depth.

Interestingly, simulations reveal that these gradients diminish in the later stages of discharge (Figure 3e). As sulfur near the separator converts to Li_2_S, surface passivation eliminates local reaction sites and inhibits further conversion in those regions. This triggers a redistribution of reaction current toward deeper portions of the cathode where active surfaces remain accessible (Figure 3f). The reaction front progressively migrates toward the cathode‐current collector interface as sulfur particles become increasingly encapsulated by a Li_2_S layer, enhancing utilization in previously underutilized regions. As a result, although steep concentration gradients form during the early stages of high‐rate discharge, the final distribution of discharge products becomes relatively uniform due to this dynamic current redistribution. The discharge process ultimately terminates when all accessible active sites are covered with Li_2_S, causing the cell voltage to fall below the cutoff threshold of 0.9 V.

To gain further insights into microstructural changes induced by electrochemical cycling, scanning electron microscopy (SEM) was performed on the 30wt.% sulfur composite electrode (Figure S4). The pristine electrode displays a relatively homogeneous distribution of sulfur‐containing particles within the SE matrix. After cycling, the electrode exhibits morphological evolution while some particles show clear changes in shape and surface features, other particles remain largely comparable to pristine morphology. It suggests that while the reaction front migrates, certain regions or particles remain kinetically isolated or become passivated before full conversion can occur. This non‐uniform morphological evolution is consistent with the spatially heterogeneous sulfur utilization and dynamically migrating reaction fronts predicted by the model.

While a higher yield of Li_2_S during discharge intuitively suggests improved sulfur utilization and greater capacity, this very outcome proves counterproductive during charging. The extensive formation of Li_2_S leads to severe passivation, which significantly hinders the reversibility of SSLS cells. This issue is examined in detail in the following section.

During charging, Li_2_S in the cathode is typically converted back to sulfur via Li_2_S_2_ as an intermediate. However, in this work, the charge reaction is approximated as a direct Li_2_S → sulfur transformation. This asymmetry is justified under solid‐state conditions, where both ion and electron transport are limited and Li_2_S is highly passivating. As a result, the oxidation pathway is predominantly rate‐limited by the breakdown of Li_2_S, enabling a simplified one‐step representation without significant loss of physical fidelity. A detailed thermodynamic and kinetic justification of this assumption, along with its rate‐dependent limitations, is provided in Section S2.

**FIGURE 4:**
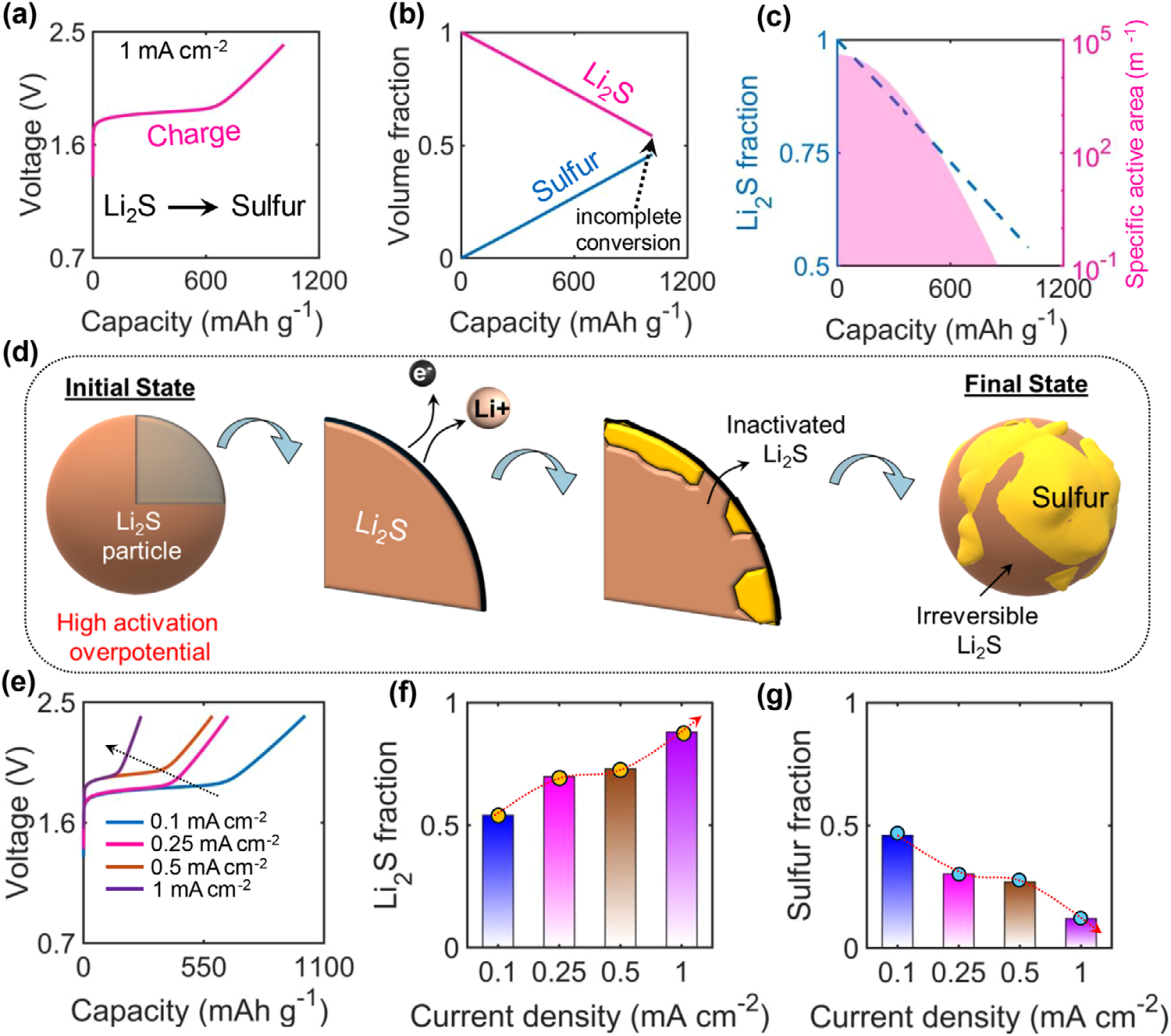
Simulation results showing (a) charging profile of the SSLS cell at 0.1 mA cm^−2^; (b) species volume fractions during charging, revealing incomplete reconversion of Li_2_S to sulfur; (c) decline in Li_2_S content accompanied by a reduction in active interfacial area. (d) Schematic illustrating particle‐level evolution with sulfur formation and residual Li_2_S, indicating partial reversibility and phase heterogeneity. (e) Simulated voltage‐capacity curves during charging at different current densities; extracted (f) Li_2_S and (g) sulfur fractions after charging, highlighting increased irreversibility with higher current rates.

Figure 4a illustrates the simulated electrochemical behavior of the SSLS cell during charging at 0.1 mA cm^−2^. A voltage plateau is observed during this process, primarily associated with the conversion of Li_2_S back to sulfur. However, the oxidation of electrochemically inactive Li_2_S often requires substantial overpotentials to initiate the reaction. As shown in Figure 4b, while some Li_2_S is converted to sulfur, complete reconversion does not occur; approximately 54% of Li_2_S remains inactivated, resulting in a mixed Li_2_S/sulfur charged state. This incomplete transformation reflects the inherent challenge of oxidizing Li_2_S, whose insulating nature hinders charge transport and limits sulfur recovery.

As the reaction progresses, the available specific active area within the cathode decreases rapidly (Figure 4c). Around 650 mAh g^−1^, a significant loss of specific active area is observed as remaining Li_2_S becomes electrochemically inaccessible, causing a sharp increase in cell overpotential and driving the voltage to the cutoff value of 2.4 V. This behavior is consistent with experimental studies, which report a notable decline in conversion efficiency under typical operating conditions [59, 60, 61, 62]. The incomplete reconversion of Li_2_S reduces both reversibility and long‐term cycling performance in SSLS cells (Figure 4d). To mitigate this, Kim et al. [45] proposed adjusting the discharge cutoff voltage to favor the formation of a mixed Li_2_S_2_/ Li_2_S product. While this strategy may lower the overall discharge capacity, Li_2_S_2_ exhibits more favorable redox kinetics compared to Li_2_S, potentially enhancing the rechargeability of SSLS batteries.

This limitation is further exacerbated at higher charging rates. The charging capacity drops sharply from 1014 mAh g^−1^ at 0.1 mA cm^−2^ to 263 mAh g^−1^ at 1 mA cm^−2^ (Figure 4e). This decline is primarily driven by elevated reaction and kinetic overpotentials, as the sluggish conversion of Li_2_S to sulfur fails to sustain the required current. The resulting polarization inhibits further Li_2_S oxidation, leading to a substantial voltage rise and reduced charge capacity. These effects are also reflected in the current‐dependent dQ/dV curves, where a distinct shift in the charging peak toward higher potentials indicates increased kinetic resistance and polarization at elevated current densities (Figure 3d). Consequently, the unconverted Li_2_S fraction increases significantly, from 54% at 0.1 mA cm^−2^ to 87% at 1 mA cm^−2^ (Figure 4f). Correspondingly, the fraction of sulfur recovered decreases from 46% to just 13% as the current increases (Figure 4g), underscoring the severe impact of kinetic limitations on the sulfur recovery.

**FIGURE 5:**
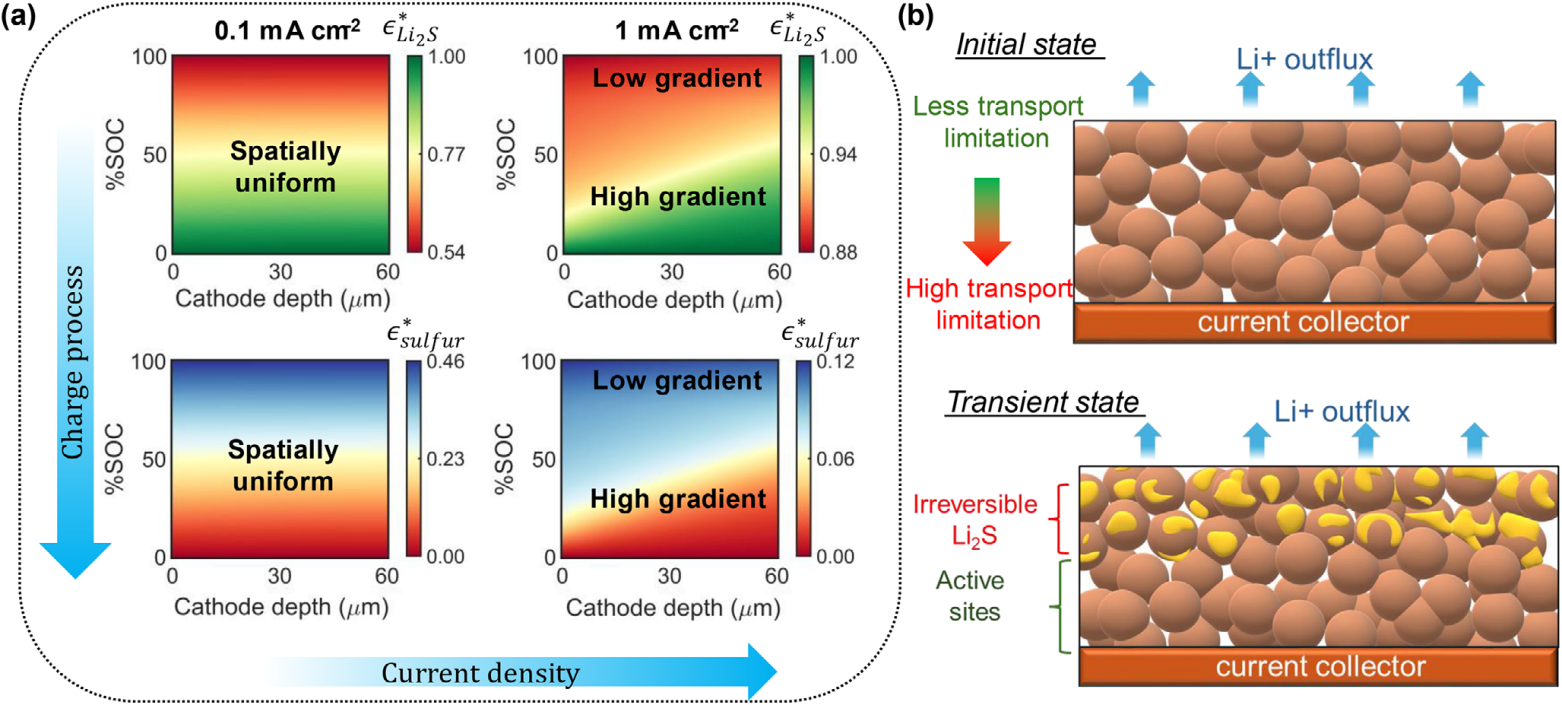
(a) Simulated spatio‐temporal distribution of Li_2_S (top) and sulfur (bottom) across the cathode depth as a function of state of charge (SOC), shown for two charge rates: 0.1 mA cm^−2^ (left) and 1 mA cm^−2^ (right). The downward direction corresponds to different charge products, while the horizontal direction compares the same species at the two charge rates. High‐rate charge leads to pronounced concentration gradients near the cathode‐separator interface, which diminish toward the end of charge due to reaction current redistribution. (b) Schematic illustrating loss of active sites near the separator‐cathode interface due to electrochemically inactive Li_2_S, leading to current redistribution within the cathode bulk.

Similar to discharging, rate‐dependent limitations significantly influence the spatial distribution of species within the cathode. At low current densities (0.1 mA cm^−2^), Li_2_S is oxidized slowly and uniformly across the cathode thickness, as the rate of Li^+^ extraction from Li_2_S keeps pace with the composite's ionic transport capacity. This enables a gradual and spatially homogeneous conversion of Li_2_S back to sulfur, with minimal concentration gradients (Figure 5a). In contrast, at high current densities (1 mA cm^−2^), the system becomes both kinetically and transport limited. In the early stages of charge, Li_2_S oxidation is most prominent near the cathode‐separator interface, leading to the rapid formation of sulfur in this region. Meanwhile, the deeper regions of the cathode remain underutilized, as the sluggish Li^+^ transport through the low‐conductivity Li_2_S network hinders activation. This results in the development of steep species concentration gradients across the electrode, with more converted sulfur near the separator side and a substantial fraction of unreacted Li_2_S toward the current collector side (Figure 5a).

As charging progresses, regions near the separator become electrochemically inactive due to the residual Li_2_S, which remains unconverted owing to the high activation overpotentials required for its oxidation. This passivation suppresses further reaction in these areas and causes the reaction current to redistribute toward deeper regions of the cathode, where unreacted Li_2_S and available interfacial pathways still exist. This shift in reaction front facilitates the continued oxidation of Li_2_S in the previously underutilized zones (Figure 5b), thereby gradually diminishing the steep concentration gradients established earlier in the process. By the end of charging, although 87% of Li_2_S remains unconverted, the spatial distribution becomes nearly uniform across the cathode, with minimal variation between the separator and current collector interfaces (86.5% vs. 87%).

Taken together, the charge and discharge analysis highlight the critical role of Li^+^ transport and reactive C|S_8_|SE interface in governing redox progression, both of which are inherently governed by cathode architecture. This influence becomes particularly apparent when examining sulfur cycling behavior. In SSLS cells, sulfur acts as the active material, with its utilization and recovery directly governing electrochemical performance and rechargeability. Higher sulfur loading reduces the SE fraction in the cathode, limiting ionic percolation pathways and creating domains where sulfur remains unutilized due to poor ion and electron accessibility.

During cathode fabrication, sulfur, SE, and carbon additives are mixed and cold‐pressed under high pressure. Despite this, voids referred to as residual or “dead” porosity persist within the electrode, restricting contact between sulfur and SE particles. This residual porosity is primarily governed by the fabrication pressure and decreases with increasing compaction. In our experiments, the cathodes were fabricated at a high compaction pressure of approximately 360 MPa and cycled under a stack pressure of 20 MPa, corresponding to a dense cathode microstructure. Accordingly, the baseline residual porosity used in the model (∼ 15%) was chosen to represent this high‐densification regime. Reducing the fabrication pressure is expected to increase residual porosity, while further increases in fabrication pressure led to progressively smaller changes as densification saturates [63]. The porosity range explored in this study (10%–30%) spans realistic fabrication conditions, from highly compacted to more loosely pressed cathodes. This residual porosity can significantly impact battery performance by affecting ion transport, stress accommodation, and degradation behavior. Building on these insights, we investigate cell performance as a function of sulfur loading and residual porosity, while maintaining a constant 10 vol% carbon content.

**FIGURE 6:**
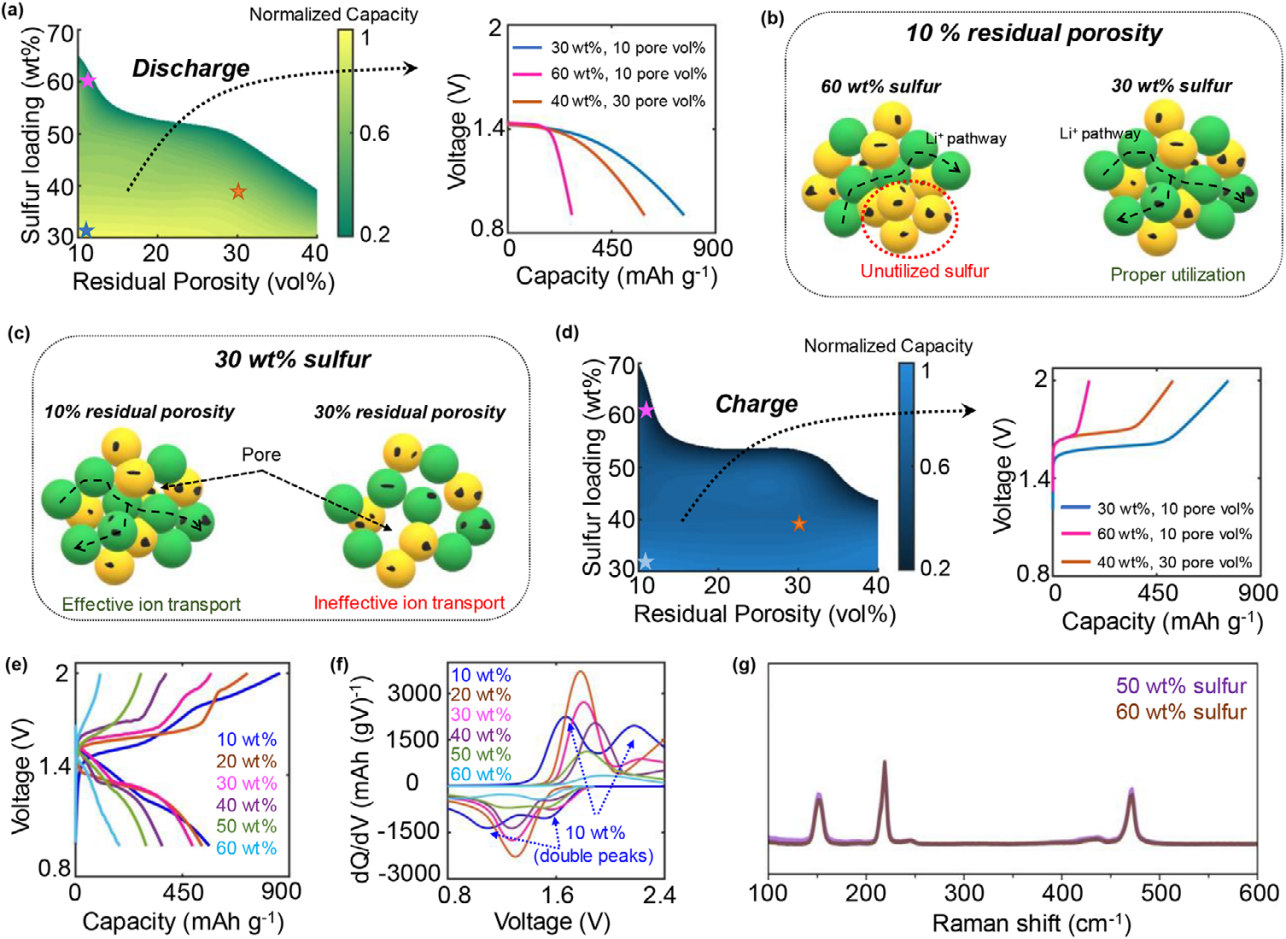
(a) Simulated normalized discharge capacity contour as a function of sulfur loading and residual porosity, with representative discharge curves. (b) Schematic illustrating that sulfur utilization at 60 wt.% loading is limited due to greater unreacted sulfur compared to 30 wt.% at 10% residual porosity. (c) Schematic illustration of ion transport efficiency for 30 wt.% sulfur at 10% and 30% residual porosity. (d) Simulated normalized charge capacity contour and representative charge curves for various sulfur‐porosity combinations. (e) Experimental discharge voltage profiles for sulfur loadings from 10 to 60 wt.%. (f) Experimental differential capacity (dQ/dV) curves exhibiting additional peaks near 2.2 and 1.2 V at low sulfur loading (high SE content), attributed to solid electrolyte degradation. (g) Raman spectra of cathodes with 50 and 60 wt.% sulfur, confirming the presence of unconverted sulfur domains.

At 10 vol% residual porosity, the discharge capacity decreases from 765 to 278 mAh g^−1^ as sulfur content increases from 30  to 60 wt.% (Figure 6a). At high sulfur loadings, the reduced SE fraction is insufficient to interface with all sulfur domains, disrupting the ionic transport network and introducing kinetic limitations that impair electrochemical performance. As a result, sulfur particles become spatially isolated from the SE (Figure 6b), leading to an increase in unutilized sulfur from 27% at 30 wt.% to 69% at 60 wt.% sulfur loading. In addition, limited SE content reduces the number of point‐to‐point connections between SE particles, further constraining Li^+^ transport within the cathode. These limitations are exacerbated by residual porosity, which interrupts essential particle contact and ion conduction pathways (Figure 6c). For instance, at 30 vol% residual porosity and 40 wt.% sulfur loading, the discharge capacity drops to 593 mAh g^−1^ and becomes negligible for sulfur loadings above 50 wt.% (Figure 6b).

A similar trend is observed during charging (Figure 6d). At 10 vol% porosity, the charging capacity declines by 79.3%, from 1014 to 209 mAh g^−1^, as sulfur loading increases from 30  to 60 wt.%. Likewise, at a fixed 30 wt.% sulfur content, increasing the residual porosity from 10 vol% to 30 vol% reduces the charging capacity to 697 mAh g^−1^, primarily due to aggravated transport limitations.

Furthermore, we experimentally investigate the charge‐discharge behavior of SSLS cells with varying sulfur content, which confirms that the utilized capacity degrades significantly with increasing sulfur loading (Figure 6e). Interestingly, an additional capacity contribution is observed at low sulfur loading (10 wt.%) and high SE content (70 wt.% LPSCl), where the voltage profiles exhibit extended sloping regions around ∼2.2 V during charging and ∼1.2 V during discharging. These features correspond to distinct peaks in the dQ/dV curves that are absent in sulfur‐rich compositions, suggesting the presence of parasitic reactions beyond the primary sulfur redox (Figure 6f). Mechanistically, the increased SE fraction results in a higher density of SE‐carbon interfaces, which are known to promote electrochemical degradation under operating voltages, especially during charging [56, 57, 58, 64, 65, 66]. This degradation arises because excess SE beyond the percolation threshold does not improve ionic conductivity but instead increases reactive interfacial area with conductive carbon and sulfur intermediates. As the sulfur content increases (50–60 wt.%), these peaks in the dQ/dV curves are suppressed, implying improved electrolyte stability, but at the cost of reduced sulfur utilization due to insufficient SE connectivity. Raman spectra of discharged cathodes further support this trade‐off: at higher sulfur contents, the spectra remain dominated by sulfur peaks, confirming the presence of unreacted sulfur domains that remain electronically or ionically isolated (Figure 6g).

These experimental findings align closely with predictions from our computational framework, further validating its ability to capture species utilization and transport limitations. However, it is important to note that our model does not incorporate SE degradation mechanisms; thus, the observed excess capacity and dQ/dV features at high SE contents, attributed to parasitic reactions, lie outside the current modeling scope. Accordingly, the sulfur‐porosity maps are constructed to capture intrinsic transport and kinetic limitations governing sulfur utilization, and do not include capacity contributions arising from SE degradation, which would otherwise artificially favor low‐sulfur compositions despite poor reversibility.

Within the scope of the present electrochemical framework, an intermediate sulfur content of 30 wt.% consistently provides the best balance between sulfur utilization and transport efficiency (Figure 6a,d). At lower sulfur contents, excessive SE exposure increases interfacial degradation and parasitic reactions, whereas at higher sulfur contents, reduced SE percolation and surface passivation lead to incomplete sulfur utilization. These competing effects give rise to a practical operating window rather than a single universal optimum. Although reduced residual porosity is favorable from a purely electrochemical perspective in the present analysis, which assumes fixed cathode geometry, the optimal porosity cannot be determined independently of chemo‐mechanical effects. When sulfur volume expansion and mechanical constraint are considered, excessively low residual porosity is expected to amplify stress localization and interfacial damage, thereby shifting the optimal porosity away from the electrochemically preferred limit. As such, identification of an optimal residual porosity requires a coupled chemo‐mechanical treatment and is beyond the scope of the current study.

## 3 Conclusion

In conclusion, this work provides a comprehensive understanding of electrochemical behavior, species evolution, and microstructural transformations during charge–discharge cycling in SSLS cells. A central limitation in these systems is the electrochemical inactivity of Li_2_S, which hinders performance and long‐term rechargeability due to its incomplete reconversion to sulfur. While prior experimental studies have reported the presence of mixed species during cycling, our framework elucidates the specific conditions under which these intermediates form and quantifies their temporal and spatial evolution within the cathode, and probes the irreversibility associated with sluggish Li_2_S oxidation kinetics. Supported by Raman spectroscopy and XRD, we systematically analyze the coupled influence of transport and kinetic limitations, particularly as governed by sulfur loading and residual porosity, to identify optimal design windows that enhance performance during both discharge and charge. Importantly, we find that low sulfur loading, while improving ionic connectivity, leads to electrolyte degradation due to an excess of reactive SE‐carbon interfaces, contributing to parasitic capacity. Conversely, high sulfur content reduces solid electrolyte percolation and results in buried, electrochemically inaccessible domains due to severe passivation. The resulting sulfur‐porosity maps provide actionable guidance for tailoring cathode architectures to achieve high sulfur utilization while mitigating transport bottlenecks. By integrating mechanistic modeling with experimental validation, this study advances the fundamental understanding of SSLS operation and delivers design principles for realizing practical, high‐energy lithium–sulfur systems. Future work will extend this framework to long‐term cycling by coupling chemo‐mechanical deformation with transport and interfacial kinetics, enabling quantitative assessment of capacity degradation arising from surface passivation, particle damage, and contact loss during repeated charge‐discharge cycles. Additionally, operando electrochemical probes, including impedance and intermittent titration techniques, will be incorporated with analysis frameworks tailored to conversion‐driven SSLS cathodes to further elucidate the evolution of kinetic and transport limitations during cycling.

## 4 Methods

### 4.1 Experimental

#### 4.1.1 Cathode Composite Preparation

Sulfur composites were prepared by ball‐milling cathode active materials (CAM), sulfur (Thermo Scientific, 325 mesh, 99.5%), Li_6_PS_5_Cl (LPSCl, Ampcera, ultrafine with ∼1 µm particle size) as the solid ionic conductor, and acetylene black powder (AB, MSE Supplies, ∼40 nm particle size). Six different sulfur‐based cathode composites were prepared, each containing 20 wt.% AB and varying sulfur content between 10 and 60 wt.%, with 10% increments. The remaining weight fraction in each composite comprised LPSC, varying between 70 and 20 wt.%. Initially, 1 g of sulfur and AB powder was manually mixed using a mortar and pestle for 15 min. This was followed by a preliminary ball milling at 500 rpm for 2 h using a Fritsch Pulverisette 7 planetary ball mill. After this step, the solid electrolyte was added to the jar, and the mixture was subjected to further milling at 500 rpm for 24 cycles, each consisting of 10 min of milling followed by 5 min of rest. All the materials were prepared in an Ar‐filled glovebox before milling to prevent moisture and oxygen exposure. After milling, the composite powders were collected and stored inside the glovebox for further use.

#### 4.1.2 Counter Electrode Fabrication

Li–In alloy counter electrodes were prepared by pressing lithium and indium foils together with a uniaxial press at 250 MPa in a 1:1 stoichiometric ratio until the resulting alloy darkened in color and became brittle. The compound was then ground using a mortar and pestle for 30 min to obtain a fine, homogeneous powder. Subsequently, 10 wt.% LPSCl was added to the mixture, and the grinding process was continued for an additional 30 min to ensure uniform distribution. The final composite anode powder was stored in an Ar‐filled glovebox.

#### 4.1.3 Solid‐State Cell Assembly

For each cell, a total of 70 mg of LPSCl powder was loaded into a 10 mm diameter polyether ether ketone (PEEK) cell and pelletized using a uniaxial press at 150 MPa for 1 min. The cathode composite (10 mg) was then placed on top of the electrolyte pellet, while the Li–In composite was loaded at the bottom. The full cell stack was subsequently pressed at 375 MPa for 5 min. A constant stack pressure of 20 MPa was applied to all assembled cells. Prior to electrochemical testing, the cells were rested for 3 h. Galvanostatic cycling was performed within a voltage window of 0.9–2.4 V vs. Li–In/In. All cell assembly and cycling procedures were conducted in an Ar‐filled glovebox using a Landt battery cycler.

#### 4.1.4 Raman Spectroscopy

Raman spectroscopy measurements were performed using a Renishaw Raman spectrometer (QONTOR Confocal Raman) equipped with a 488 nm laser operating in the visible/near‐IR range. The samples were placed between two glass slides and sealed with electrical tape to prevent air ingress. Spectra were collected at 10% laser power with 1‐s exposure time and 120 accumulations. Data analysis, including background subtraction, normalization, smoothing, and cosmic ray removal, was carried out using Wire 5.2 software.

#### 4.1.5 X‐ray Diffraction

A Rigaku Miniflex with a Cu‐Kɑ radiation source was used. The cells were disassembled and loaded onto the sample holder and coated with Kapton tape in an Ar‐filled glovebox to prevent air ingress.

#### 4.1.6 Scanning Electron Microscopy (SEM)

SEM imaging was performed using a Hitachi SU8230 microscope operated at an accelerating voltage of 10 kV and a working distance of 8 mm. Cell samples were carefully sectioned using a scalpel to expose the electrode cross‐sections. During transfer and loading into the SEM chamber, the samples were exposed to ambient atmosphere for less than 10 s, followed by evacuation of the loading chamber for approximately 30 s.

## Conflicts of Interest

The authors declare no conflicts of interest.

## Supporting information




**Supporting File**: advs73922‐sup‐0001‐SuppMat.docx.

## Data Availability

The data that support the findings of this study are available from the corresponding author upon reasonable request.
