# Supplementary material for: Passivation‐Induced Species Dynamics and Microstructural Evolution in Solid‐State Lithium–Sulfur Cathodes
Source: Adv Sci (Weinh). 2026 Jan 26;13(30):e20537. doi: 10.1002/advs.202520537 (PMC13248834; doi:10.1002/advs.202520537)
Supplement: Supplementary file 1 — Supporting File: advs73922‐sup‐0001‐SuppMat.docx. [file ADVS-13-e20537-s001.docx]

**Supporting Information**

**Passivation-Induced Species Dynamics and Microstructural Evolution in Solid-State Lithium-Sulfur Cathodes**

*Arpan K. Sharma^1^, Bairav S. Vishnugopi^1^, Elif Pınar Alsaç^2^, Matthew T. McDowell^2,3^, and Partha P. Mukherjee^1,^* ^*^

^1^School of Mechanical Engineering, Purdue University, West Lafayette, IN 47907, USA

^2^George W. Woodruff School of Mechanical Engineering, Georgia Institute of Technology, Atlanta, GA, USA.

^3^School of Materials Science and Engineering, Georgia Institute of Technology, Atlanta, GA, USA.

^*^*Correspondence*: pmukherjee@purdue.edu

**S1. Computational framework to predict electrochemical performance**

**S1.1. Reaction mechanism**

Based on existing literature and corroborated by Raman spectroscopy (**Figure 2f**, main manuscript), Li_2_S_2_ is considered the sole intermediate product in the solid-solid conversion reaction at the cathode.

At the anode, lithium undergoes oxidation:

$Li\to Li^{+}+e^{-}$ (S1)

Li⁺ is transported across the cell via point-to-point contacts between solid electrolyte (SE) particles, while electrons travel through the conductive carbon network due to the poor electronic conductivity of sulfur (S_8_). During discharge, the cathodic reaction proceeds via a two-step reduction:

$S_{8}+8Li^{+}+8e^{-}\to4Li_{2}S_{2}$ (S2)

$Li_{2}S_{2}+2Li^{+}+2e^{-}\to2Li_{2}S$ (S3)

During charging, Li_2_S oxidation is modeled as a single-step reaction. This simplification is justified by the dominant kinetic limitation associated with Li_2_S oxidation and the likely transient presence of Li_2_S_2_. This assumption preserves the model’s fidelity in reproducing experimental voltage profiles and sulfur utilization trends (See Model validation in **S.1.4**). The reaction at the cathode is described as follows:

$8Li_{2}S\to16Li^{+}+{16e}^{-}+S_{8}$ (S4)

To simplify the formulation while retaining mechanistic relevance, the following assumptions are made:

1. Sulfur and SE particles are represented as monodisperse spheres in point contact with each other.
2. All electrochemical processes occur under isothermal conditions.
3. Volumetric expansion/contraction and associated mechanical stresses arising from Li_2_S_2_ and Li_2_S formation are neglected.

**S1.2. Computation of effective microstructural properties.**

The microstructures for SSLS cell cathode are generated stochastically using the commercial tool GeoDict,^[1–5]^ and the effective microstructural properties, like tortuosity, electronic conductivity and specific active area, are calculated using Direct Numerical Simulations (DNS) as discussed below.^[1,5]^

**Tortuosity**

Tortuosity describes the resistance offered by the pore phase to the ions while moving through the pore network. The tortuosity calculation involves solving steady state concentration balance in the pore phase,^[5]^ given by:

$\nabla.\left( D\nabla C \right)=0$ (S5)

Tortuosity is a directional property, hence it is calculated in all three x,y,z directions. To estimate in x direction, Dirchilet boundary conditions are applied at the left and right xy planes. The concentration at left face is set to C = 0, and at right face is set to C = 1. This ensures that net flow is in one direction. On the remaining faces, Neumann boundary conditions are applied, $\frac{\partial C}{\partial n}=0$, to make sure the flow does not leak through those faces. The solution obtained by integrating the concentration gradient over one of the extreme faces gives the estimated flux. Then the corresponding tortuosity can be obtained using the equation given below:

$j_{x}= -D\frac{\epsilon}{\tau_{x}} \left[ \frac{C\left( x_{right} \right)-C\left( x_{left} \right)}{x_{right}-x_{left}} \right]= -D\frac{\epsilon}{\tau}[\frac{1}{L_{x}}]$ (S6)

, where ε is the SE volume fraction, τ is the tortuosity, D is the bulk diffusivity in the solution phase and $j_{x}$ is the flux in x-direction. It must be noted that bulk diffusivity is set to 1 for SE phase, and 0 for sulfur, voids and carbon additives. Tortuosity in y and z directions can be computed by setting the boundary conditions suitably as mentioned above.

The mean tortuosity in the SE phase is then given by:

$\tau_{mean}=\frac{1}{3}(\tau_{x}+\tau_{y}+ \tau_{z})$ (S7)

**Electronic conductivity**

The effective electronic conductivity is determined by solving Laplace’s equation for the electric potential:

$$\nabla.\left( \sigma\nabla\phi\right)=0$$

Here, the conductivity σ is set to 10^6^ mS cm^-1^ within the carbon additive phase, and 0 in both the pore and SE phases. To compute the dimensionless effective electronic conductivity in the x-direction (σ_eff,x_), boundary conditions are applied such that ϕ = 1 at the inlet face, ϕ = 0 at the outlet face, and no-flux conditions on all other faces. The effective conductivity σ_x_ is then calculated using:

$\sigma_{x}=\frac{\left( \int_{y-z plane} \sigma\frac{\partial\phi}{\partial x}\left. \right|_{x=0}dydz \right)}{(\frac{\phi_{x=L_{x}}-\phi_{x=0}}{L_{x}})}$ (S8)

Analogous calculations are performed for the y and z directions to obtain σ_y_ and σ_z_. The average dimensionless electronic conductivity is given by:

$\sigma_{mean}=\frac{1}{3}(\sigma_{x}+\sigma_{y}+ \sigma_{z})$ (S9)

To verify that the estimated transport properties are independent of domain size, the full workflow-from microstructure generation to transport property evaluation-is repeated for multiple domain lengths, defined as L = n × R_max_, where R_max_ is the radius of the largest AM or SE particle and n takes values of 6, 8, 10, 12, and 14. The voxel size is fixed at $\Delta=\frac{R_{min}}{10}$, with R_min_ being the radius of the smallest particle. Additionally, a sensitivity study is conducted by varying Δ while keeping the domain size fixed at L = 10 × R_max_. The results show that once the domain and voxel resolutions satisfy $\frac{L}{R_{max}}=10$ and $\frac{R_{min}}{\Delta}=10$, the tortuosity and consequently the effective transport properties stabilize, indicating grid-size independence. These values are thus adopted in all DNS for microstructural property calculations.

**Specific active area**

In composite cathodes, electrochemical reactions occur at the solid-solid interfacial contact between the sulfur and SE phases. The active surface area at this interface is estimated using a modified version of the Minkowski method,^[6,7]^ as shown in the following equation:

$a_{s}=\frac{4\pi I_{S-SE}}{M_{x}M_{y}M_{z}\Delta}.f$ (S10)

In this context, I_S-SE_ represents the number of cell faces that have sulfur and SE cells on either side. M_x_, M_y_ and M_z_ denote the total number of cells in the x, y, and z directions, respectively, while Δ represents the voxel length. The term 𝑟=𝑁Δ refers to the digitized sphere’s radius, where I_N_, representing the number of surface faces, approaches the ideal sphere as the number of surface faces increases. The parameter $f=\frac{N^{2}}{I_{N}}$​ is derived based on the convergence of the digitized sphere’s surface area toward that of an ideal sphere, which is 4𝜋𝑟^2^. This method is applicable to any geometry, enabling the computation of the interfacial area between sulfur and SE phases.

**S1.3. Electrochemical Performance Model**

The computed microstructural descriptors are integrated into a physics-informed framework to predict the charge-discharge behavior of SSLS cells. The complex, non-linear electrochemical response of these systems stems from the coupled interplay of interfacial reaction kinetics, ionic transport through the solid electrolyte, and spatially heterogeneous species evolution within the composite cathode. These phenomena span multiple length scales, from the particle level to the porous electrode scale, and are governed by the following set of conservation and reaction equations, which form the basis of the electrochemical performance model developed in this study.

**Discharging**

1. Charge conservation:

Charge conservation in the SE and Acetylene Black (AB) particle can be expressed as:

$\nabla i_{SE}+\nabla i_{AB} = 0$ (S11)

, where i is the current density in both the phases.

The electric potential in the solid phase (sulfur particles) at cathode can be determined using Ohm’s law as follows:

$\nabla.\left( \sigma_{s}^{eff} \nabla\phi_{s} \right) -a_{s}i_{AB} = 0$ , and (S12)

The electric potential in the solid electrolyte is governed by the gradient of ionic concentration, as follows:

$\nabla.\left( \kappa_{s}^{eff} \nabla\phi_{e} \right) +\nabla.\left( \kappa_{D}^{eff} \nabla\ln c_{e} \right)+a_{s}i_{SE} = 0$ (S13)

, where $\sigma$, $\kappa_{s}^{eff}$, $\kappa_{D}^{eff}$, c_e_ and $a_{s}$ are electrical conductivity of the AB particle,^[5]^ ionic conductivity of SE particle, diffusive ionic conductivity of Li^+^, concentration of Li^+^ and specific active area respectively. Since, we have assumed the concentration of Li+ ions within the pore space to be constant (assumption 2), the equation (6) reduces to:

$\nabla.\left( \kappa_{s}^{eff} \nabla\phi_{e} \right)+a_{s}i_{SE} = 0$ (S14)

It must be noted that Porous Electrode Theory is used to calculate the effective transport properties in both the phases in the porous cathode,^[8]^ as shown below:

$\kappa^{eff} = \kappa\left( \frac{\varepsilon}{\tau_{mean}} \right)$ (S15)

, where $\varepsilon$and $\tau$ are the porosity and tortuosity of cathode respectively.

1. Electrochemical kinetics:

The charge transfer reaction rate is dependent on the volume fraction on the sulfur, Li_2_S_2_ and Li_2_S, and hence are modeled using Butler-Volmer equation as below:

$i_{1} = n_{1}Fk_{1}\left\{ \varepsilon_{S_{8}}^{*}\exp\left( \beta_{1} \frac{F}{RT} \eta_{1} \right) -\varepsilon_{Li_{2}S_{2}}^{*}\exp\left( -\left( 1-\beta_{1} \right) \frac{F}{RT} \eta_{1} \right) \right\}$ (S16)

$\eta_{1} = \phi_{S} - \phi_{E}-E_{1}^{0}$ (S17)

$i_{2} = n_{2}Fk_{2}\left\{ \varepsilon_{Li_{2}S_{2}}^{*}\exp\left( \beta_{2} \frac{F}{RT} \eta_{2} \right) -\varepsilon_{Li_{2}S}^{*}\exp\left( -\left( 1-\beta_{2} \right) \frac{F}{RT} \eta_{2} \right) \right\}$ (S18)

$\eta_{2} = \phi_{S} - \phi_{E}-E_{2}^{0}$ (S19)

, where k_j_ is the reaction rate coefficient, $\varepsilon_{i}^{*}$ is the normalized volume fraction of solid species in cathode, E^0^_j_ is the reactions standard potential vs Li/Li^+^, $\eta_{j}$ is the overpotential, and $\beta_{j}$ is the symmetry factor for the reactions.

The normalized volume fractions of solid species are calculated based on the initial volume fraction of sulfur as follows:

$\varepsilon_{S_{8}}^{*}=\frac{\varepsilon_{S_{8}}}{\varepsilon_{S_{8},0}}$ (S20)

$\varepsilon_{Li_{2}S_{2}}^{*}=\frac{\varepsilon_{Li_{2}S_{2}}\times V_{S_{8}}}{\varepsilon_{S_{8},0}\times4\times V_{Li_{2}S_{2}}}$ (S21)

$\varepsilon_{Li_{2}S}^{*}=\frac{\varepsilon_{Li_{2}S}\times V_{S_{8}}}{\varepsilon_{S_{8},0}\times8\times V_{Li_{2}S}}$ (S22)

, where ε_i_ is the volume fraction of the i-th species, $\varepsilon_{S_{8},0}$ is the initial volume fraction of elemental sulfur, and $V_{S_{8}}$, $V_{Li_{2}S_{2}}$, and $V_{Li_{2}S}$ are the molar densities of sulfur, Li_2_S_2_ and Li_2_S, respectively.

To maintain charge neutrality, the model must implicitly ensure that the sum of the current densities (i_1_ and i_2_) equals the applied current density (**Figure S1(a)**).

$i_{1}+i_{2}=I_{app}$ (S23)

The values of $E_{1}^{0}$​ and $E_{2}^{0}$​ were estimated by observing the prominent redox peaks in the differential capacity (dQ/dV vs V) curves.

1. Species conservation:

As the discharge continues, sulfur converts to Li_2_S_2_, and ultimately to Li_2_S. The rate of the conversion is dependent on the rate of reaction as follows:

$\frac{\partial\varepsilon_{S_{8}}}{\partial t} = -\frac{a_{s}i_{1}}{n_{1}F}V_{S_{8}}$ (S24)

$\frac{\partial\varepsilon_{Li_{2}S_{2}}}{\partial t} = a_{s}\left( \frac{4i_{1}}{n_{1}F} - \frac{i_{2}}{n_{2}F} \right)V_{Li_{2}S_{2}}$ (S25)

$\frac{\partial\varepsilon_{Li_{2}S}}{\partial t} = -a_{s}\frac{2i_{2}}{n_{2}F}V_{Li_{2}S}$ (S26)

, where ε_i_ is the volume fraction of the i-th species, V_j_ represents the molar densities of the solid species, and n_1_ and n_2_ represent the number of electrons transferred in reactions (S2) and (S3) respectively.

1. Surface passivation:

The final discharge product, Li_2_S, is ionically and electronically insulating and forms a passivating layer over sulfur particles, thereby suppressing further electrochemical reactions at the interface. As Li_2_S accumulates at the surface, it reduces the specific electrochemically active area available for reaction. To capture this dynamic, the model includes a passivation penalty term that links the active area to the local surface volume fraction of Li_2_S.

The specific active area a_s_ is modeled as a decay function of the normalized Li_2_S surface coverage, with the rate of decay dependent on the applied current density. This formulation accounts for the experimentally observed phenomenon that higher current densities accelerate Li_2_S accumulation and earlier onset of surface passivation.

$a_{s}=\frac{a_{s}^{0}}{1+e^{\alpha}}$ (S27)

$\alpha=\beta(s,I_{app})\times\epsilon_{Li_{2}S,threshold}^{*}$ (S28)

, where $a_{s}^{0}$ is the initial specific active area, $\varepsilon_{Li_{2}S, threshold}^{*}$is the reference threshold volume fraction of Li_2_S beyond which surface blockage begins to dominate. $\beta$is a fitting parameter that governs the influence of current rate (I_app_, Am^-2^) and sulfur loading (s, wt%) on the passivation threshold, reflecting how faster cycling promotes earlier onset of Li_2_S-induced surface isolation. Based on values calibrated to experimental data, β is described by the following correlation, valid for 30-60 wt % sulfur and 0.1 to 1 mA cm^-2^ (1-10 Am^-2^), with a R^2^ ­value of 0.99:

$\ln\beta=52.36-29.22\times\ln s+0.0364\times\ln I_{app}+4.4\times\left( \ln s \right)^{2}+0.265\times\left( \ln I_{app} \right)^{2}+0.123\times(\ln s)\times(\ln I_{app})$ (S29)

**Charging**

1. Charge conservation**:**

The charge conservation equation during the charging is similar to the discharging process. Since the direction of the current is reversed during the charging behavior, hence equations are modified as follows:

$\nabla.\left( \sigma_{s}^{eff} \nabla\phi_{s} \right) +a_{s}i_{AB} = 0$, and (S30)

$\nabla.\left( \kappa_{s}^{eff} \nabla\phi_{e} \right)-a_{s}i_{SE} = 0$ (S31)

1. Electrochemical kinetics:

Since charging is assumed as a single step kinetics, the reaction-kinetic equations are reduced to:

$i = nFk_{\mathrm{ch}}\left\{ \varepsilon_{\mathrm{Li}_{2}S}^{*}\exp\left( \beta_{\mathrm{ch}} \frac{F}{RT} \eta_{\mathrm{ch}} \right) -\varepsilon_{S_{8}}^{*}\exp\left( -\left( 1-\beta_{\mathrm{ch}} \right) \frac{F}{RT} \eta_{\mathrm{ch}} \right) \right\}$ (S32)

$\eta_{\mathrm{ch}} = \phi_{S} - \phi_{E}-E_{ch}^{0}$ (S33)

, where k_ch_ is the reaction rate coefficient, $\varepsilon_{i}^{*}$ is the normalized volume fraction of solid species in cathode, E^0^_ch_ is the reactions standard potential vs Li/Li^+^, $\eta_{ch}$ is the overpotential, and $\beta_{ch}$ is the symmetry factor for charging process.

The normalized volume fractions for solid species are modified using the initial volume fraction of Li_2_S as follows:

$\varepsilon_{Li_{2}S}^{*}=\frac{\varepsilon_{Li_{2}S}}{\varepsilon_{Li_{2}S_{,0}}}$ (S34)

$\varepsilon_{S_{8}}^{*}=\frac{\varepsilon_{S_{8}}\times V_{Li_{2}S}\times8}{\varepsilon_{Li_{2}S,0}\times V_{S_{8}}}$ (S35)

The values of $E_{ch}^{0}$​ was estimated by observing the prominent redox peak in the differential capacity (dQ/dV vs V) curves.

1. Species conservation:

As the charging process continues, Li_2_S gets consumed and gets converted to sulfur. The concentration of the solid species at any time is given as follows:

$\frac{\partial\varepsilon_{Li_{2}S}}{\partial t} = -a_{s}\frac{8i}{nF}V_{Li_{2}S}$ (S36)

$\frac{\partial\varepsilon_{S_{8}}}{\partial t} = a_{s}\frac{i}{nF}V_{S_{8}}$ (S37)

, where ε_i_ is the volume fraction of the i-th species, V_j_ represents the molar densities of the solid species, and n represents the number of electrons transferred during the reaction process.

1. Surface passivation:

Just like discharge process, the specific active area available for the charging process also decreases as the reaction continues. However, as discussed in the manuscript, entire Li_2_S does not convert to sulfur fully, hence the area is reduced as elemental sulfur forms and decreases sharply after a critical value of sulfur is formed. The equation to capture this behavior is given as:

$a_{s}=\frac{a_{s}^{0}}{1+e^{\alpha^{2.1}}}$ (S38)

$\alpha=\delta(s,I_{app})\varepsilon_{S_{8}, threshold}^{*}$ (S39)

, where $\varepsilon_{S_{8}, threshold}^{*}$ is the the reference threshold volume fraction of elemental sulfur, beyond which further oxidation becomes kinetically constrained. $\delta$is a fitting parameter that governs the influence of current rate and sulfur loading on the passivation threshold, reflecting how accelerated cycling promotes earlier onset of Li_2_S-induced surface isolation and limits the availability of reactive interfaces during charge. Based on values calibrated to experimental data, $\delta$ is described by the following correlation, valid for 30-60 wt % sulfur and 0.1 to 1 mA cm^-2^ (1 - 10 Am^-2^) , with a R^2^ value of 0.98:

$\ln\delta=39.71-21.72\times\ln s-0.007\times\ln I_{app}+3.18\times\left( \ln s \right)^{2}+0.254\times\left( \ln I_{app} \right)^{2}-0.064\times(\ln s)\times(\ln I_{app})$ (S40)

**Conservation of sulfur in cathode**

Because sulfur does not exit the electrochemical system but instead transitions between different phases, its total amount is implicitly conserved throughout the numerical simulations (**Figure S1(b)**). The total sulfur content in the cathode at any given time is computed using the following relation:

$S_{total}= \int_{L_{sep}}^{L_{sep}+L_{cat}} (\frac{\epsilon_{S_{8}}}{V_{S_{8}}}+\frac{\epsilon_{Li_{2}S_{2}}}{4V_{Li_{2}S_{2}}}+\frac{\epsilon_{Li_{2}S}}{8V_{Li_{2}S}})dx$ (S41)

The coefficients 1, 1/4, and 1/8 account for the number of sulfur atoms per formula unit in S_8_, Li_2_S_2_ and Li_2_S, respectively. This formulation ensures strict mass conservation of sulfur across the electrode thickness.


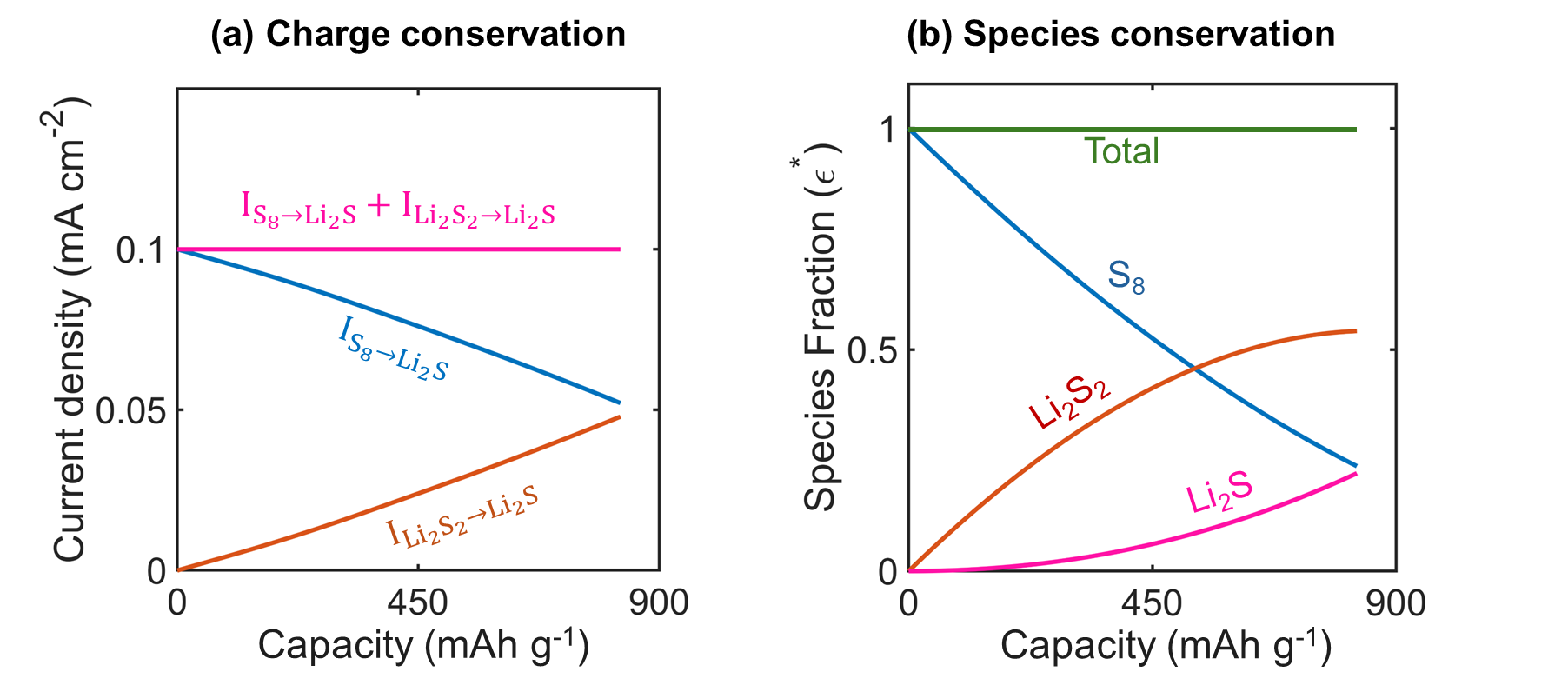


**Figure S1**: (a) The sum of the reaction current densities is equal to the applied current density. (b) Conservation of total sulfur content during the discharge process, confirming sulfur mass balance within the system.

**S1.4. Model Validation**

The proposed electrochemical framework was comprehensively validated against experimental data through a two-pronged approach: (i) rate-dependent validation across current densities, and (ii) composition-dependent validation across sulfur loadings. Specifically, simulations were compared with experimental charge-discharge profiles for cathodes with 30 wt%, 40 wt%, and 60 wt% sulfur content, cycled at current densities ranging from 0.1 to 1 mA cm^-2^ (1-10 A m^-2^). These conditions span both kinetic and transport-limited regimes and allow us to rigorously evaluate model fidelity. **Figure S2** illustrates the voltage-capacity profiles at four different current densities for a 30 wt% sulfur cathode. The model successfully captures key features of the voltage response, rate-dependent overpotentials, and the sharp rise near cutoff. Final discharge and charge capacities show excellent agreement, with mean errors of 1.46% and 1.27%, respectively (**Table S1**). **Figure S3** extends the validation to three different sulfur loadings (30, 40, and 60 wt%) at a fixed current density of 0.1 mA cm^-2^. The model reproduces the suppression of capacity and increase in polarization with higher sulfur content, accurately reflecting the role of ionic percolation and interfacial accessibility. Quantitative comparison (**Table S2**) yields a mean absolute error of 5.87% in discharge and 3.69% in charge capacities.

Overall, the strong agreement across both rate and compositional regimes underscores the robustness of the mechanistic framework and its ability to capture passivation, spatial species evolution, and microstructure-dependent electrochemical behavior in SSLS cells.

***
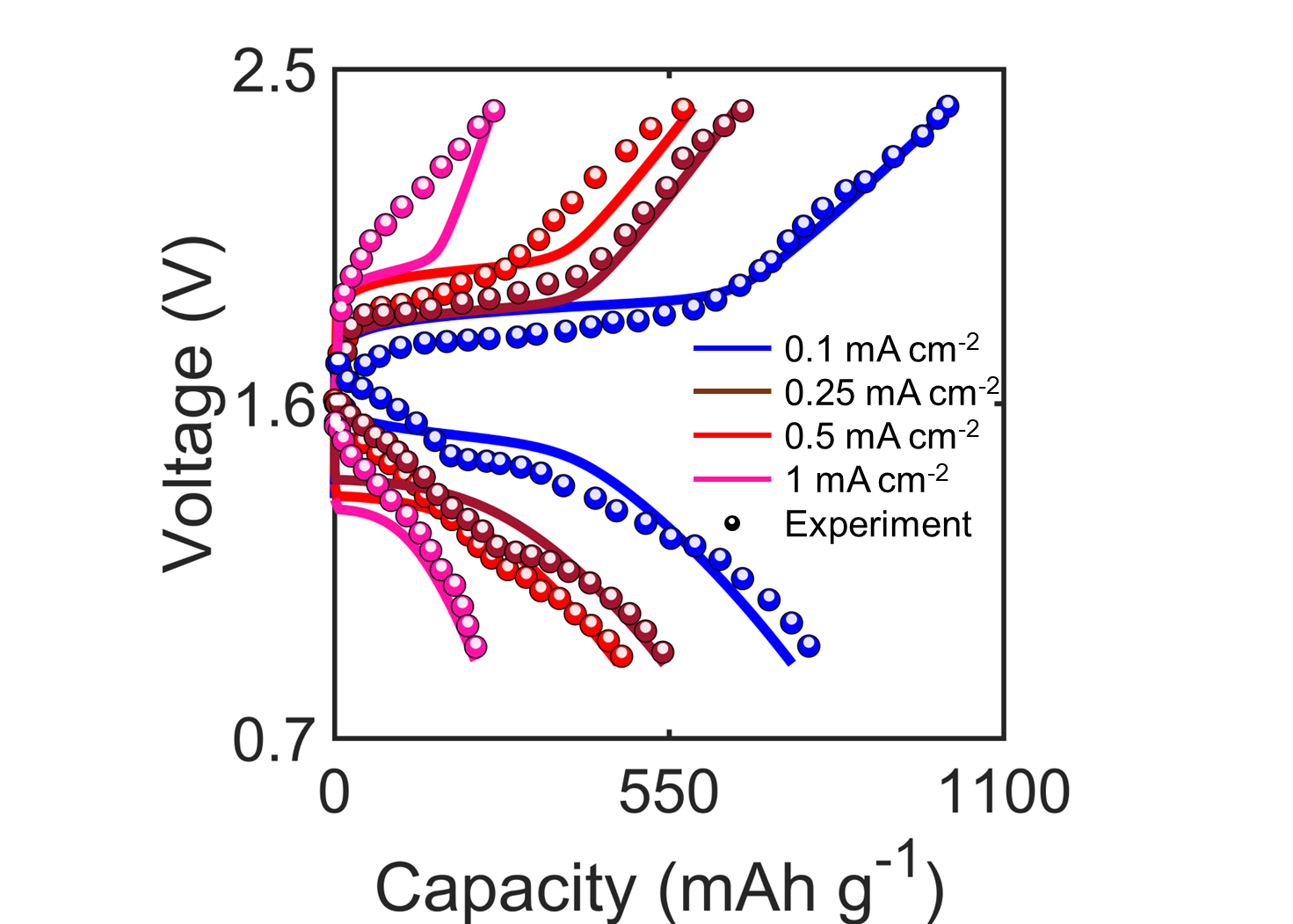
***

**Figure S2**. Comparison of the charge and discharge behavior as predicted by the electrochemical model at four different current densities of 0.1, 0.25, 0.5 and 1 mA cm^-2^ for a 30 wt% sulfur loading cathode against the experimental values.

**Table S1:** Summary of final capacities (in mAh g^-1^) for various current densities at 30 wt% sulfur loading as outlined in experimenal and simulation results

| **Current density**  **(mA cm^-2^)** | **Discharge** | | | **Charge** | | |
| --- | --- | --- | --- | --- | --- | --- |
|  | **Experiment** | **Model** | **\|Error\|**  **(%)** | **Experiment** | **Model** | **\|Error\|**  **(%)** |
| 0.1 | 779 | 752 | 3.47 | 1008 | 1014 | 0.57 |
| 0.25 | 539 | 542 | 0.51 | 670 | 662 | 1.10 |
| 0.5 | 471 | 466 | 1.12 | 572 | 590 | 3.13 |
| 1 | 231 | 233 | 0.75 | 261 | 262 | 0.28 |

**
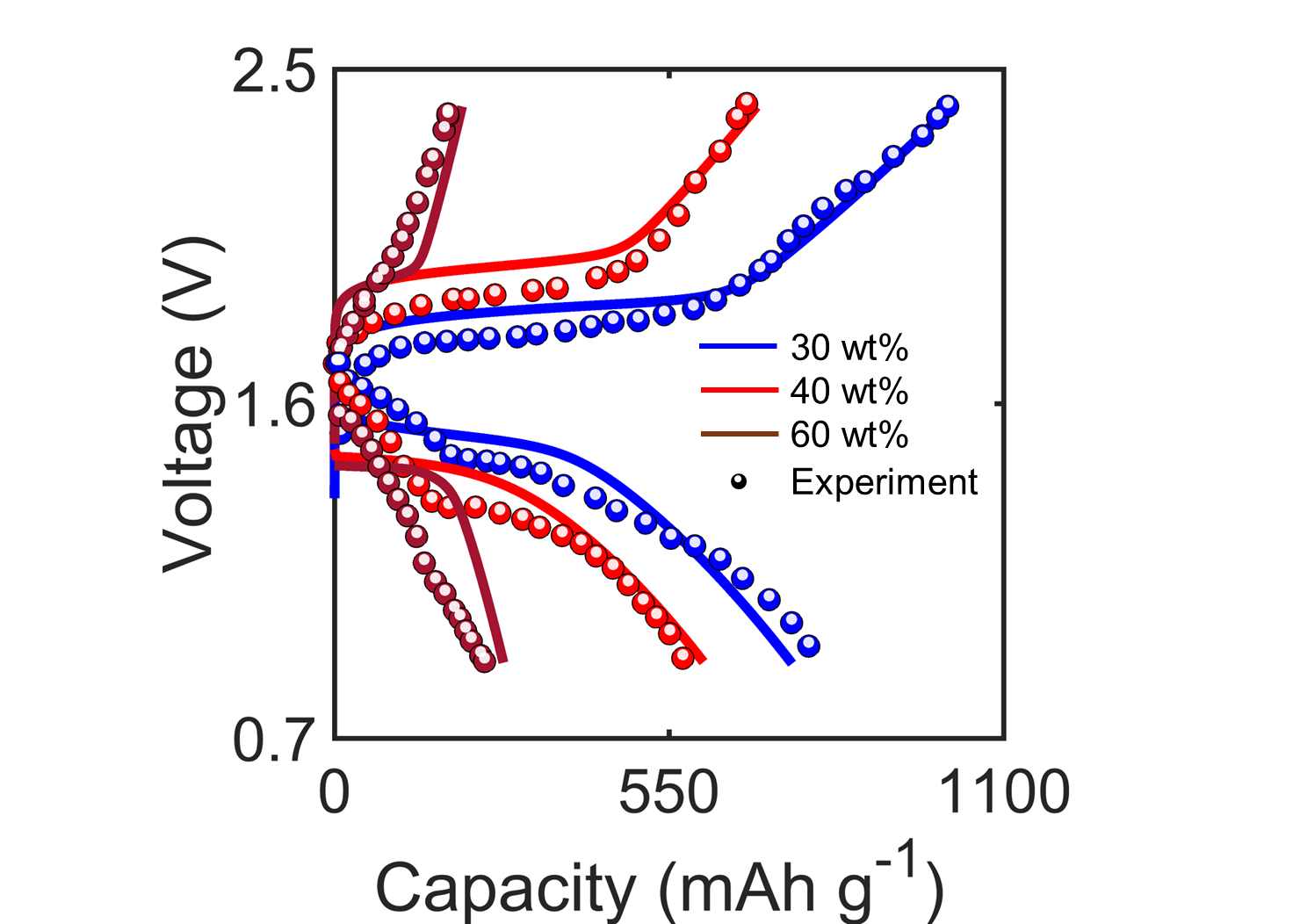
**

**Figure S3**. Comparison of the charge and discharge behavior as predicted by the electrochemical model at three different sulfur loadings of 30, 40 and 60 wt% at a current density of 0.1 mA cm^-2^ against the experimental values.

**Table S2:** Summary of final capacities (in mAh g^-1^) for various sulfur loading cathodes at a current density of 0.1 mA cm^-2^ as outlined in experimenal and simulation results

| **Sulfur loading**  **(wt%)** | **Discharge** | | | **Charge** | | |
| --- | --- | --- | --- | --- | --- | --- |
|  | **Experiment** | **Model** | **\|Error\|**  **(%)** | **Experiment** | **Model** | **\|Error\|**  **(%)** |
| 30 | 779 | 752 | 3.47 | 1008 | 1014 | 0.57 |
| 40 | 572 | 609 | 6.42 | 677 | 694 | 2.5 |
| 60 | 246 | 265 | 7.72 | 186 | 201 | 7.99 |

**S1.5.** **Boundary Conditions for Electrochemical Performance Model**

The boundary conditions employed in the electrochemical performance model are summarized in **Table S3**. A Dirichlet boundary condition is imposed at the cathode-current collector interface for the solid electrolyte potential, 𝜙_E_, which is essential to avoid ill-posedness in the elliptic partial differential equations governing charge conservation. ^[9,10]^ Model parameters are listed in **Table S4**, and the initial conditions used for numerical convergence are provided in **Table S5**. Simulations proceed until any of the criteria defined in equations (S16), (S18), and (S23) are satisfied, or until the cell voltage reaches the defined cutoff value.

**Table S3.** Boundary conditions for the electrochemical model.

| Equation | Anode-separator boundary (x = 0) | Separator-cathode boundary (x = L_sep_) | Cathode-current collector (x = L_sep_ + L_cat_) |
| --- | --- | --- | --- |
| Solid electrolyte phase potential | $\frac{\partial\phi_{e}}{\partial x}=\frac{I_{app}}{A}$ | $\left( \kappa_{s}^{eff} \nabla\phi_{e} \right)_{L_{sep}- \delta}= \left( \kappa_{s}^{eff} \nabla\phi_{e} \right)_{L_{sep} + \delta}$ | $\frac{\partial\phi_{e}}{\partial x}=0$  $\varphi_{e}=0$* |
| Solid sulfur phase potential | $\sigma_{s}^{eff} \frac{\partial\phi_{s}}{\partial x}= 0$ | $\frac{\partial\phi_{s}}{\partial x}=0$ | $\sigma_{s}^{eff} \frac{\partial\phi_{s}}{\partial x}= -\frac{I_{app}}{A}$ |
| *Dirichlet boundary condition to ensure solution of elliptical partial differential equations | | | |

**Table S4**. Parameters used in electrochemical model.

| **Parameter** | **Value** | **Unit** | **Symbol** |
| --- | --- | --- | --- |
| **Cathode Properties** | | | |
| Pristine Porosity | 0.15 | - | ε_0_ |
| Electronic conductivity | 10 | S m^-1^ | σ |
| Length of cathode | 62 | µm | L_cat_ |
| Sulfur particle diameter | 5 | µm | D |
| Density of sulfur | 2.07 ×10^6^ | g m^-3^ | $\rho_{S_{8}}$ |
| Density of SE | 2 ×10^6^ | g m^-3^ | $\rho_{Li_{2}S_{2}}$ |
| Density of carbon | 1.9 ×10^6^ | g m^-3^ | $\rho_{Li_{2}S}$ |
| **Separator Properties** | | | |
| Length of separator | 250 | µm | L_sep_ |
| **Electrochemical Properties - Discharge** | | | |
| Reaction (1) standard potential vs Li/Li^+^ | 1.5 | V | $E_{1}^{0}$ |
| Reaction (2) standard potential vs Li/Li^+^ | 1.45 | V | $E_{2}^{0}$ |
| Reaction (1) Symmetry factor | 0.5 | - | $\beta_{1}$ |
| Reaction (2) Symmetry factor | 0.5 | - | $\beta_{2}$ |
| Cutoff Li_2_S volume fraction | 1 | - | $\varepsilon_{Li_{2}S, threshold}^{*}$ |
| Cutoff voltage | 0.9 | V | V_dis,cut_ |
| **Electrochemical Properties - Charge** | | | |
| Reaction standard potential vs Li/Li^+^ | 1.75 | V | $E_{ch}^{0}$ |
| Reaction symmetry factor | 0.5 | - | $\beta_{ch}$ |
| Cutoff S_8_ volume fraction | 1 | - | $\varepsilon_{S_{8}, threshold}^{*}$ |
| Cutoff voltage | 2.4 | V | V_ch,cut_ |
| **Kinetic Properties - Discharge** | | | |
| Discharge reaction (1) rate constant | 5×10^-4^ | mol (s-m^2^)^-1^ | k_1_ |
| Discharge reaction (2) rate constant | 5×10^-5^ | mol (s-m^2^)^-1^ | k_2_ |
| Intrinsic ionic conductivity of solid electrolyte | 0.5 | mS cm^-1^ | $\kappa$ |
| **Species properties** | | | |
| Molar density of sulfur | 1.232×10^-4^ | m^3^ mol^-1^ | $V_{S_{8}}$ |
| Molar density of Li_2_S_2_ | 2.955×10^-5^ | m^3^ mol^-1^ | $V_{Li_{2}S_{2}}$ |
| Molar density of Li_2_S | 1.477×10^-5^ | m^3^ mol^-1^ | $V_{Li_{2}S}$ |

**Table S5**. Initial conditions for model parameters used in the electrochemical model.

| **Parameter** | **Initial value** |
| --- | --- |
| $\varphi_{s}$ | 1.8 V |
| $\varphi_{e}$ | 0 V |
| $\varepsilon_{Li_{2}S_{2}}^{*}$ | 1×10^-15^ |
| $\varepsilon_{Li_{2}S}^{*}$ | 1×10^-15^ for discharge |
| $\varepsilon_{S_{8}}^{*}$ | 1×10^-15^ for charge |
| Δt | 1 s |


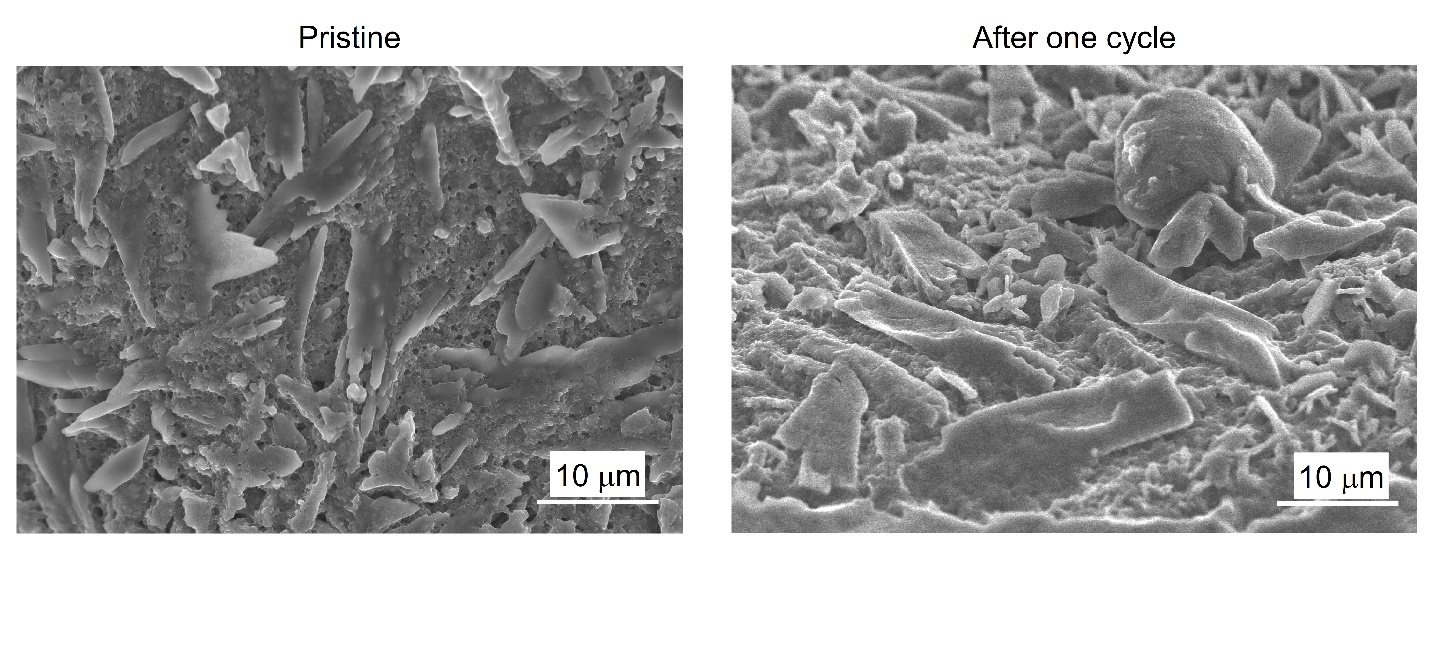


Figure S4. SEM images of the 30 wt% sulfur cathode in the pristine state (left) and after one charge-discharge cycle (right), showing heterogeneous morphological evolution after cycling.

**S2. Rationale for assuming a single-step reaction pathway during charging**

In the present modeling framework, the discharge process is described using a multi-step pathway that explicitly resolves Li_2_S_2_ as an intermediate, whereas the charging process is simplified as a single-step Li_2_S → S reaction. This asymmetric treatment is a deliberate modeling choice motivated by the thermodynamic stability and kinetic behavior of sulfur species under solid-state conditions.

First-principles calculations reported by Kim et al.^[11]^ show that Li_2_S is thermodynamically more stable than Li_2_S_2_, with a formation energy of approximately -1.59 eV atom^-1^ for Li_2_S compared to -1.01 eV atom^-1^ for Li_2_S_2_. This substantial stability difference indicates that Li_2_S represents the most energetically favorable discharge product, while Li_2_S_2_ is comparatively metastable. In addition, Li_2_S oxidation is widely reported to be kinetically sluggish due to its poor ionic and electronic conductivity, whereas Li_2_S_2_ exhibits higher redox activity and lower energetic barriers for conversion.

As a result, the charging process in SSLS cathodes is predominantly governed by the initial breakdown of electrochemically inactive Li_2_S. Any Li_2_S_2_ formed during charging is therefore expected to be transient and to convert further toward sulfur without significant accumulation. Under these conditions, explicitly resolving Li_2_S_2_ during charging has a limited impact on the dominant reaction bottleneck but substantially increases model complexity.

At elevated charging rates, partial accumulation of intermediate species may become more pronounced and contribute to deviations between simulated and experimental voltage responses. Explicit inclusion of Li_2_S_2_ during charging is therefore expected to improve quantitative agreement under high-rate conditions but is not anticipated to alter the qualitative trends in sulfur recovery and irreversibility captured by the present framework.

**References**

[1] C.-F. Chen, A. Verma, P. P. Mukherjee, *J. Electrochem. Soc.* **2017**, *164*, E3146.

[2] Math2Market GmbH, *GeoDict User Guide 2022*, Math2Market GmbH, DE, **2021**.

[3] B. S. Vishnugopi, A. Verma, P. P. Mukherjee, *J. Electrochem. Soc.* **2020**, *167*, 090508.

[4] A. Bielefeld, D. A. Weber, J. Janek, *J. Phys. Chem. C* **2019**, *123*, 1626.

[5] A. N. Mistry, K. Smith, P. P. Mukherjee, *ACS Appl. Mater. Interfaces* **2018**, *10*, 6317.

[6] M. Parmananda, C. Norris, S. A. Roberts, P. P. Mukherjee, *ACS Appl. Mater. Interfaces* **2022**, *14*, 18335.

[7] K. G. Naik, B. S. Vishnugopi, P. P. Mukherjee, *ACS Appl. Mater. Interfaces* **2022**, *14*, 29754.

[8] J. Newman, W. Tiedemann, *AIChE Journal* **1975**, *21*, 25.

[9] E. Kreyszig, *Advanced Engineering Mathematics*, Wiley, Hoboken, NJ, **2006**.

[10] M. D. Greenberg, *Advanced Engineering Mathematics*, Upper Saddle River, N.J. : Prentice Hall, **1998**.

[11] J. T. Kim, A. Rao, H.-Y. Nie, Y. Hu, W. Li, F. Zhao, S. Deng, X. Hao, J. Fu, J. Luo, H. Duan, C. Wang, C. V. Singh, X. Sun, *Nat Commun* **2023**, *14*, 6404.
